# Gain enhancement wideband CPW antenna based on artificial magnetic conductor

**DOI:** 10.1038/s41598-025-89622-9

**Published:** 2025-02-28

**Authors:** May AboEl-Hassan, A. E. Farahat, K. F. A. Hussein

**Affiliations:** Microwave Engineering Department, Electronics Research Institute (ERI), Cairo, Egypt

**Keywords:** Artificial magnetic conductor, Metasurface, Frequency selective surface, Wideband antenna, Planar monopole antenna, Engineering, Mathematics and computing

## Abstract

Unidirectional radiation and, hence, gain enhancement can be achieved by placing a primary radiator (simple antenna) at suitable height over a reflector to diminish back radiation and to enhance the forward radiation. The reflector used to enhance the gain is usually an electrically conducting surface (ECS) or an artificial magnetic conducting surface (AMCS). The ECS unifies the direction of radiation by reflecting the incident wave with $180^\circ$ phase shift, which requires the placement of the antenna at large enough height above the reflector to avoid destructive interference between the incident and the reflected waves. The AMCS is a metasurface constructed as periodic structure to produce reflection with 0° phase. This allows the antenna to be placed near the AMCS without destructive interference. Thus, the combined structure of the antenna and the AMCS reflector can have lower profile than that resulting in the case of employing ECS. The present work proposes a planar wideband antenna as well as an AMCS to produce unidirectional radiation with high gain over a wide frequency band. The proposed antenna is a planar octagon-shaped monopole patch with inverted U-slot and is fed through a coplanar waveguide (CPW). Both the radiating patch and the feeding line are printed on a single-sided substrate of type Rogers RT5880 of dimensions $27\,\text{mm}\times 37\,\text{mm}$ and thickness $1.57\,\text{mm}$. The patch geometry is designed to maximize the radiation efficiency by cutting an inverted U-shaped slot with long base. The proposed AMCS consists of $5\times 5$ cells and has dimensions $70\,\text{mm }\times 70\,\text{mm}$. The metallic patches of AMCS cells are printed on the top layer of a substrate of type Rogers’ RO4003C of thickness $1.52\,\text{mm}$. Both the proposed antenna and AMCS are fabricated for experimental evaluation of the performance of the radiating structure. It is shown by simulation and measurement that the proposed antenna when based on the proposed AMCS produces a realized gain of $11.5\,\text{dBi}$ and total efficiency of greater than $80\%$ over the frequency band 3.5–6.5 GHz.

## Introduction

The rapidly growing demands for reliable, large-capacity, and high-speed wireless communication networks makes the perfect antenna design a crucial requirement. Low profile antennas that offer high gain, wide frequency band, and unidirectional radiation are particularly of important interest for the recent research work^1,2^. The gain of omnidirectional antennas, such as monopole and dipole antennas, can indeed be enhanced in a specific direction by using metal reflectors^3^. This method effectively reduces radiation in other directions, thus increasing the antenna gain without necessarily increasing its physical size, which is a significant advantage over antenna arrays. Periodic structures like the electromagnetic band gap designs and metasurfaces can play a key role in enhancing the gain of antennas. Such surfaces are mostly used as reflectors that are placed very near to a primary radiating element to reflect the incident signal in phase-agreement, which leads to a cumulative superposition of radiation from the primary antennas^4,5^. These periodic structures essentially act as mirrors for electromagnetic waves^6–8^.

A lot of research work has been interested in the use of artificial magnetic conducting surface (AMCS) of various geometries to be employed as a reflector that improves the performance of wideband antennas with a reduced profile^3–5,9^. For example, in^1^ a novel $6\times 6$-cell AMCS is proposed for the purpose of enhancing the gain of a dipole antenna with reduced profile. A maximum gain up to 9.9 dBi is achieved with maximum cross polarization level of less than $-30\text{ dB}$. In^2^, an umbrella-shaped radiating element is constructed using the intersection of two ellipses. This antenna provides a wide impedance bandwidth of $10.35\text{ GHz}$. In^4^, a novel cross-slot AMC (CSAMC) is proposed to work as a reflector that achieves $\pm 90^\circ$ reflection phase bandwidth of $4.07\text{ GHz}$ ($44.69\%$). In^5^, an ultra-wide band (UWB) monopole antenna CPW-fed based on compact coplanar frequency selective (FSS) array is proposed to produce enhanced gain. In^10^, two layers of FSS reflectors consisting of $7\times 5$ crossed elements on the lower and upper surfaces a dielectric substrate are proposed for gain enhancement over wide frequency band.

In this paper, a wideband planar monopole patch antenna of octagonal shape and U-slot is designed to operate over the frequency band 3.5–6.3 GHz band. The antenna fed through a coplanar waveguide (CPW) feeder to produce wideband operation and omnidirectional radiation. A wideband AMCS is designed to as metasurface of periodic structure to produce resonance ($0^\circ$-phase reflection) at $7.44$ GHz. The proposed AMCS is used as a reflector that is placed on one side of the printed monopole antenna to produce unidirectional radiation and to enhance its gain. The structure of the proposed AMCS consists of 5 $\times 5$ unit cells are designed on the top surface of a dielectric substrate with a solid ground plane on the other face. The AMCS is separated from the radiating antenna by an air gap. The proposed printed monopole antenna can be used alone to produce omnidirectional radiation or it can be placed at an appropriate distance over the proposed AMCS reflector to produce unidirectional radiation and enhanced gain.

The present paper is organized in ten sections after the introduction. Section “Antenna design and principle of operation” presents the proposed wideband planar antenna design. Section “Experimental work of the antenna in free space” describes the experimental work for investigating the performance of the proposed antenna. Section “Surface current distribution for the antenna” provides illustrations for the surface current distribution on the radiating monopole patch antenna. Section “Radiation characteristics of the antenna in free space” presents the radiation patterns of the proposed antenna when placed in free space. Section “AMC reflector design” describes the design of the unit cell of the proposed AMCS. Section “Performance of the monopole patch antenna when placed over AMCS” presents the numerical results concerned with the change of the radiation characteristics of the wideband antenna when placed over the proposed AMCS. Section “Experimental investigation of the effects of the AMCS on the antenna performance” describes the experimental work concerned with the practical investigation of the radiating structure composed of the planar antenna when placed over the AMCS. Section “Comparing the effects of the ECS and AMCS on the antenna performance” presents important numerical results for comparison between the effects of the proposed AMCS and an ECS of the same dimensions when both of them are employed to enhance the antenna performance. Section “Summary of the proposed antenna performance” provides some comparisons with recently published related work. Section “Conclusion” summarized the conclusions of the present work.

## Antenna design and principle of operation

This section presents the proposed antenna design. The principles of wideband operation considering the impedance matching and antenna efficiency are explained.

### Antenna geometry and structure

**Fig. 1:**
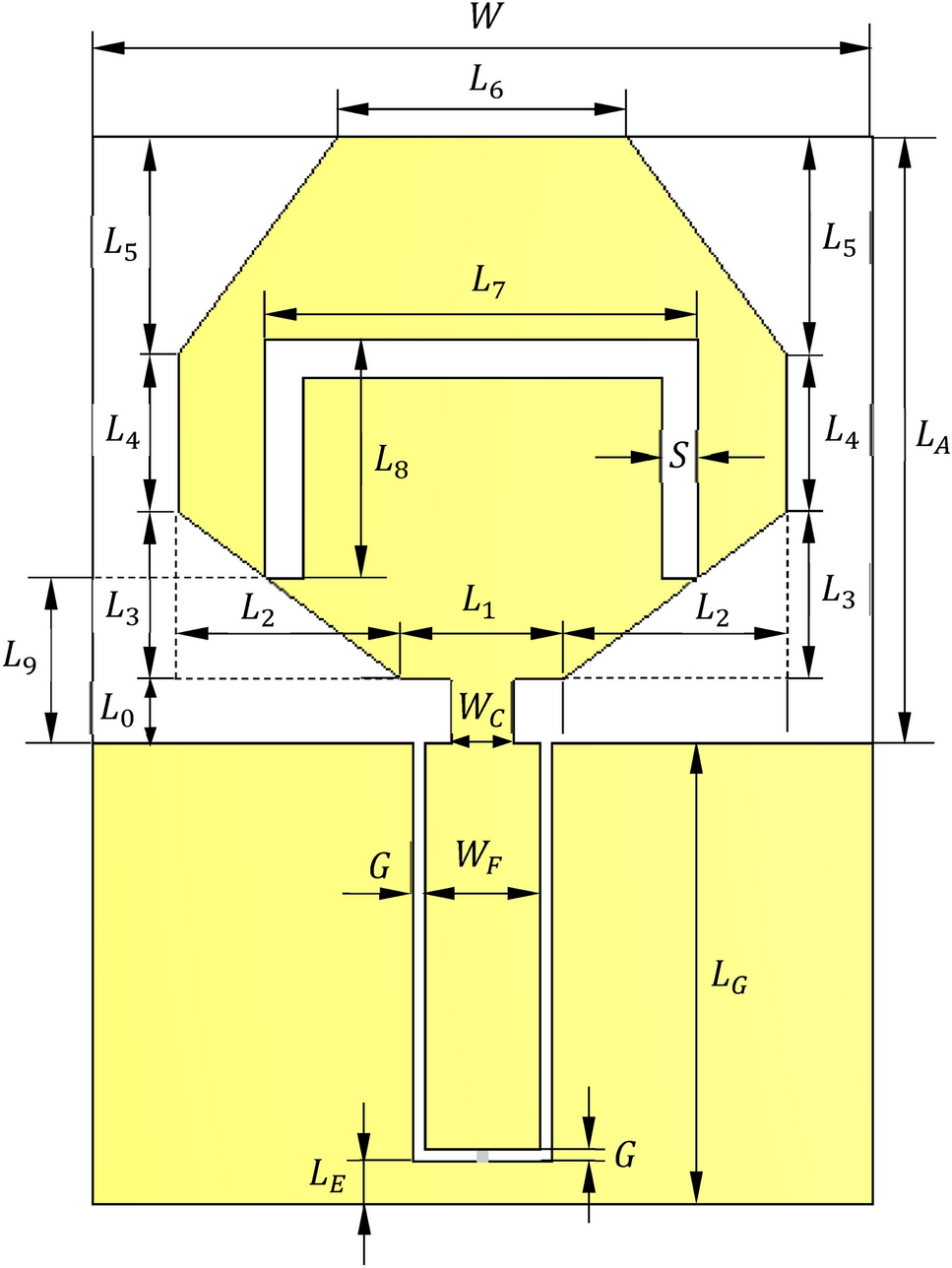
Geometry of the proposed planar monopole antenna.

The geometry of the proposed planar antenna is shown in Fig. 1. The radiating element is a planar octagon-shaped monopole patch with inverted U-slot. The monopole patch is fed through a CPW. Both the radiating patch and the feeding line are printed on a single-sided substrate of type Rogers RT5880 of dielectric constant ${\varepsilon }_{r}= 2.2$ and los tangent $\text{tan}\delta =0.0009$. The substrate has dimensions $27\,\text{mm}\times 37\,\text{mm}$ and thickness, $h=1.57\,\text{mm}$. The patch geometry is designed to maximize the radiation efficiency by cutting an inverted U-shaped slot with long base. The presence of this slot results in an edge current with very high surface density flowing along the slot edges. This, in turn, enhances the electric field in the slot, thereby leading to increase the radiation efficiency over wide range of the frequency.

### Design of the CPW feeder

The feeding CPW region is designed to match the antenna impedance to $50\Omega$ source over a wide frequency band. The CPW has a central strip of width ${W}_{F}$ and side slots of width $G$. The characteristic impedance of the quasi-TEM mode of the CPW is expressed as follows^11^.

1 $${Z}_{0}=\frac{30\pi }{\sqrt{{{\varepsilon }_{r}}_{eff}}} \frac{K\left({\acute{k}}_{0}\right)}{K\left({k}_{0}\right)}$$

The effective dielectric constant, ${{\varepsilon }_{r}}_{eff}$, of the quasi-TEM mode of the CPW can be expressed as follows^11^.

2 $${{\varepsilon }_{r}}_{eff}=1+\frac{{\varepsilon }_{r}-1}{2} \frac{K\left({\acute{k}}_{0}\right)}{K\left({k}_{0}\right)} \frac{K\left({k}_{1}\right)}{K\left({\acute{k}}_{1}\right)}$$

where ${\varepsilon }_{r}$ is the dielectric constant of the substrate material and $K$ denotes the complete elliptic integral of the first kind, which is defined as follows.

3 $$K\left(k\right)={\int }_{0}^{\frac{\pi }{2}}\frac{d\theta }{\sqrt{1-k{\text{sin}}^{2}\theta }}$$

The arguments, ${k}_{0}$, ${\acute{k}}_{0}$, ${k}_{1}$, and ${\acute{k}}_{1}$, of $K$ are defined as follows.

4 $${k}_{0}=\frac{G}{G+2{W}_{F}}, {\acute{k}}_{0}=\sqrt{1-{k}_{0}^{2}}, {k}_{1}=\frac{\text{sinh}\left(\pi G/4h\right)}{\text{sinh}\left[\pi \left(G+2{W}_{F}\right)/4h\right]}, {\acute{k}}_{1}=\sqrt{1-{k}_{1}^{2}}$$

The characteristic impedance of the CPW can be set to ${Z}_{C}=50\Omega$ by properly setting the values of the strip and slot widths (${W}_{F}$ and $G$, respectively) according to (1).

Both the values of ${Z}_{C}$ and the length, ${L}_{G}$, of CPW region are determined to achieve impedance matching over the required frequency band. This is performed through the CST simulator using parameter sweeps and design optimization to get the values of the of the CPW design parameters (${W}_{F}$, $G$, ${L}_{G}$) that realize impedance matching over the widest frequency band. A parametric study is performed to get the best values of the antenna geometrical parameters presented in Fig. 1 to achieve the highest performance of the proposed antenna. Some examples of such parametric studies are presented in Section “Antenna design and principle of operation”. The best dimensions of the radiating patch and the feed line are listed in Table 1.

**Table 1:** Dimensions of the proposed antenna.

Dimension	$W$	${L}_{A}$	${L}_{0}$	${L}_{1}$	${L}_{2}$	${L}_{3}$	${L}_{4}$	${L}_{5}$	${L}_{6}$
Value (mm)	$27$	$21$	$2.2$	$5.5$	$7.75$	$5.8$	$5.5$	$7.5$	$10$
Dimension	${L}_{7}$	${L}_{8}$	${L}_{9}$	${L}_{E}$	${L}_{G}$	${W}_{C}$	${W}_{F}$	$S$	$G$
Value (mm)	$15$	$8.3$	$5.7$	$1.5$	$16$	$2.2$	$4$	$1.3$	$0.4$

### Parametric study for final antenna design

**Fig. 2:**
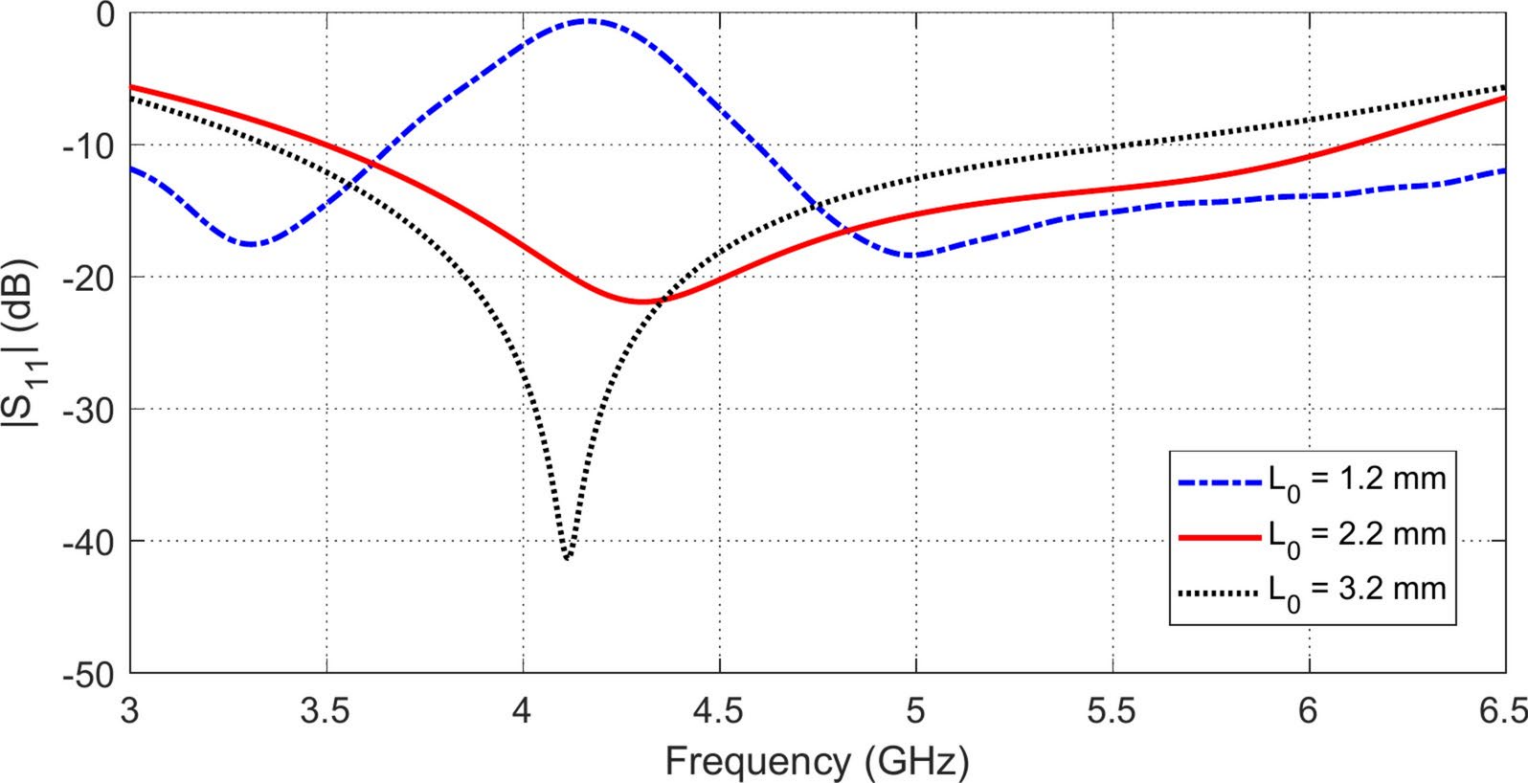
Variation of the frequency behavior of $\left|{S}_{11}\right|$ with varying the length ${L}_{0}$, of the strip connecting the central strip of the CPW feeder to the octagonal patch.

**Fig. 3:**
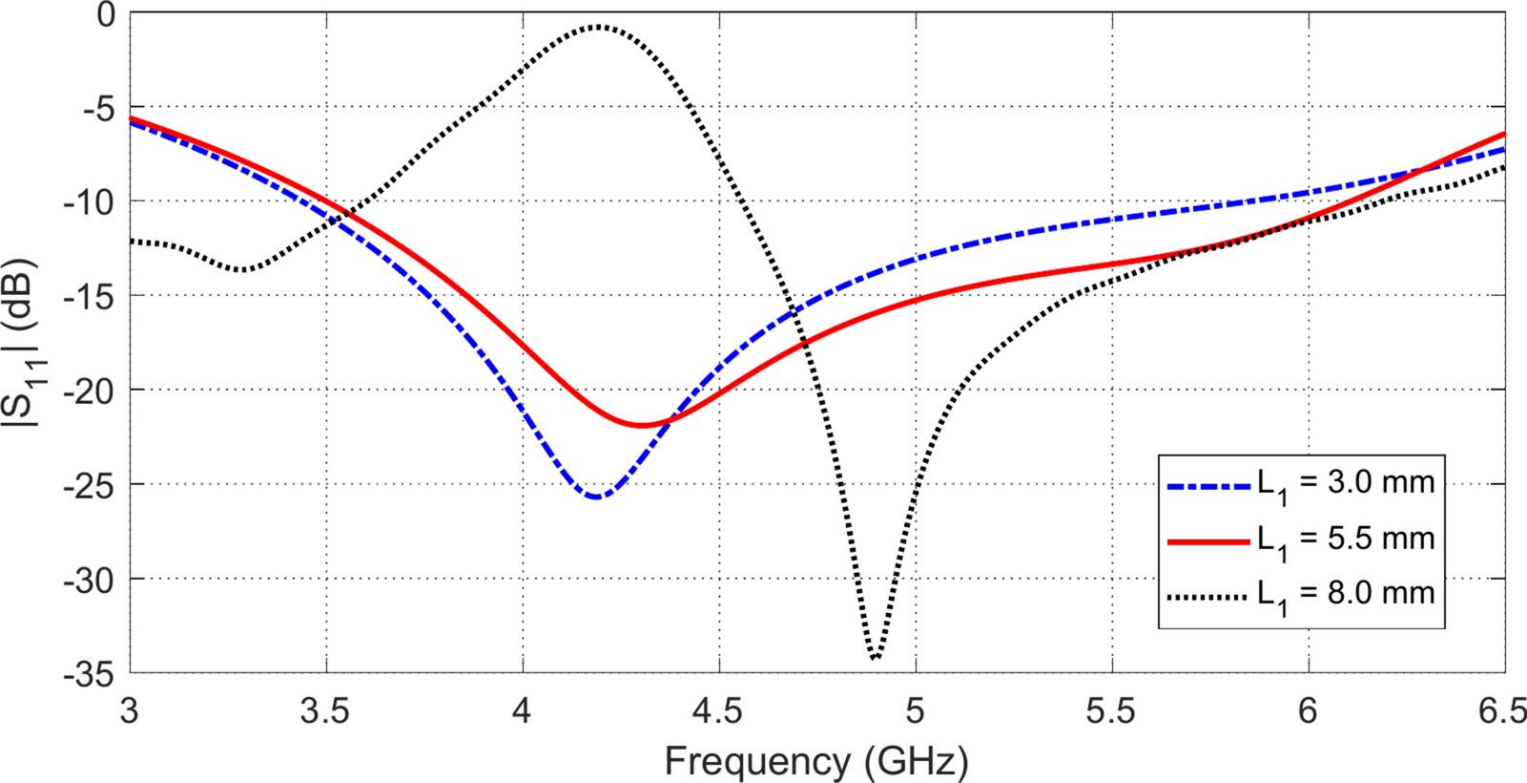
Variation of the frequency behavior of $\left|{S}_{11}\right|$ with varying the length ${L}_{1}$ of the horizontal side of the octagonal patch at the point of feeding.

**Fig. 4:**
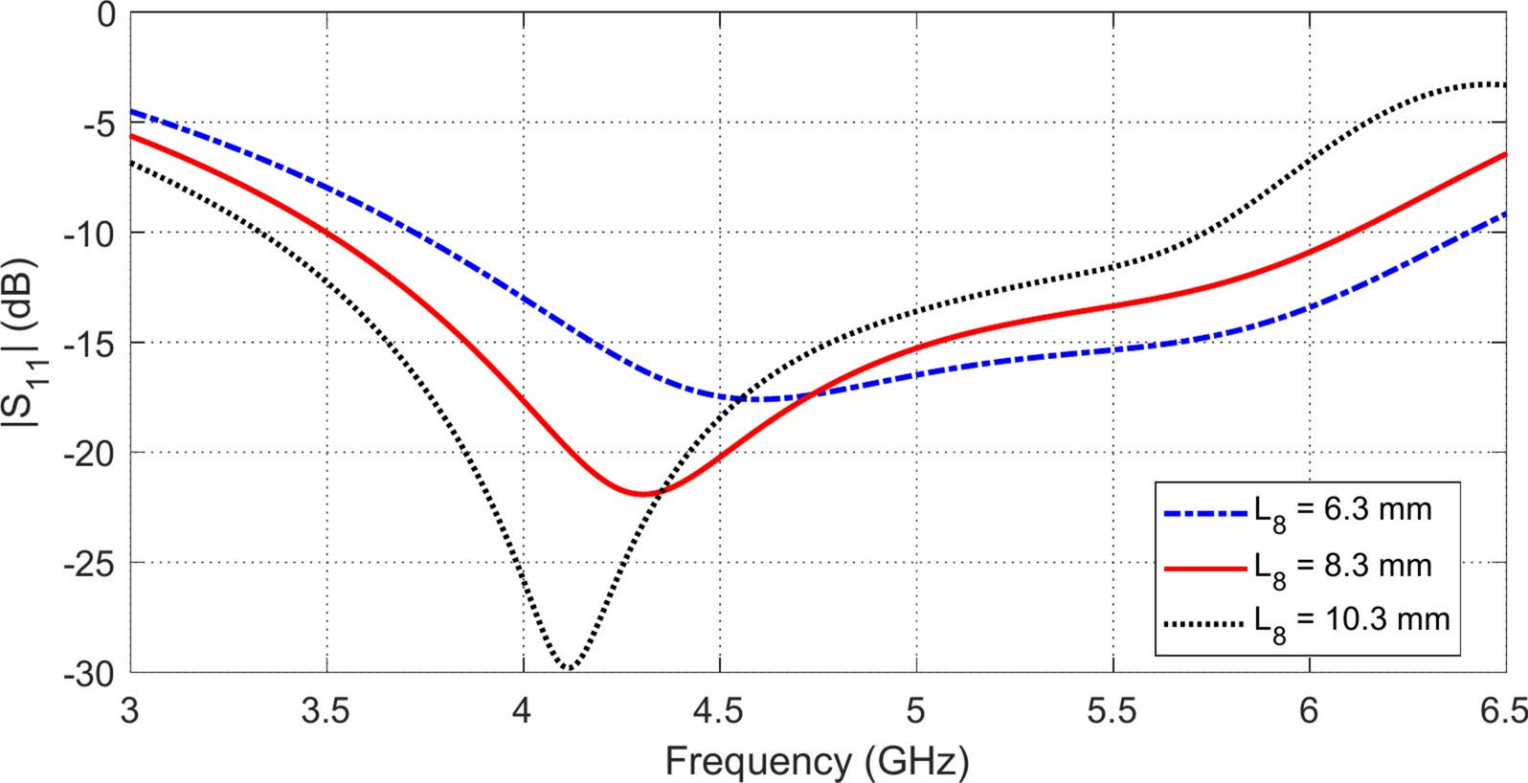
Variation of the frequency behavior of $\left|{S}_{11}\right|$ with varying the length ${L}_{8}$ of the vertical sides of the inverted U-shaped slot.

**Fig. 5:**
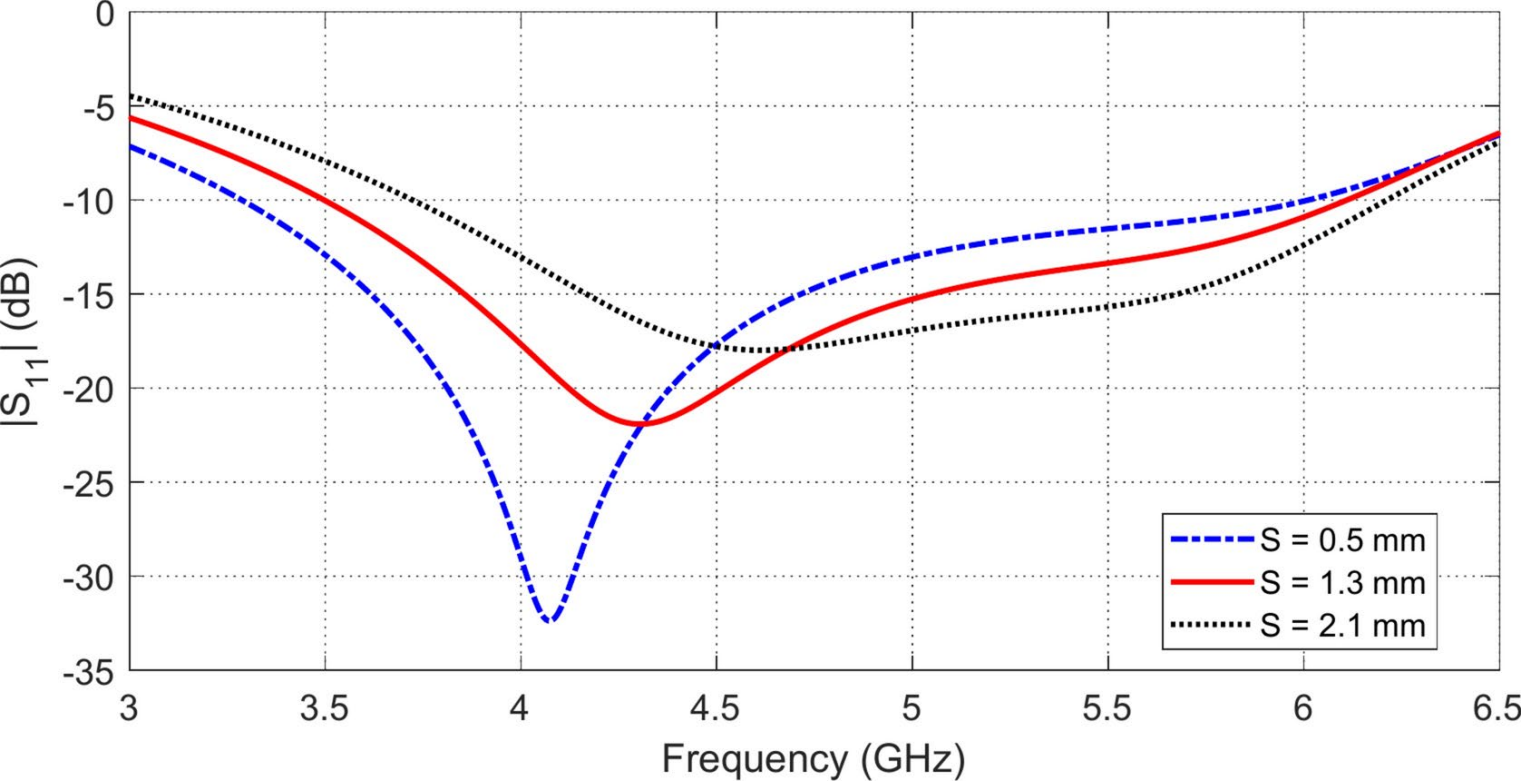
Variation of the frequency behavior of $\left|{S}_{11}\right|$ with varying the slot width $S$.

The geometrical design of the proposed antenna structure shown in Figure l is subjected to parametric study by the CST simulator to get the optimum dimensions of the radiating patch and the CPW feeder. For example, varying the length, ${L}_{0}$, of the strip connecting the central strip of the CPW feeder to the hexagonal patch affects the frequency response of $\left|{S}_{11}\right|$ as shown in Fig. 2. The most critical dimension that affects the antenna impedance matching is the length, ${L}_{1}$, of the horizontal side of the octagonal patch at the point of feeding. The variation of ${L}_{1}$ affects the frequency response of $\left|{S}_{11}\right|$ as shown in Fig. 3. The variation of the length, ${L}_{8}$, of the vertical sides of the U-shaped slot affects the frequency response of $\left|{S}_{11}\right|$ as shown in Fig. 4. The slot width, $S$, has a considerable effect on the distribution of the electric field in the slot and, consequently, the distribution of the electric current density over the long edges of the U-shaped slot. The frequency response of $\left|{S}_{11}\right|$ is affected by varying $S$ as shown in Fig. 5. It is shown that the desired frequency band ($3.5-6.0\,\text{ GHz}$) of impedance matching is satisfied when the four design parameters investigated above are set to ${L}_{0}=2.2\,\text{mm}$, ${L}_{1}=5.5\,\text{mm}$, ${L}_{8 }=8.3\,\text{mm}$, and $S=1.3\,\text{mm}$.

## Experimental work of the antenna in free space

**Fig. 6:**
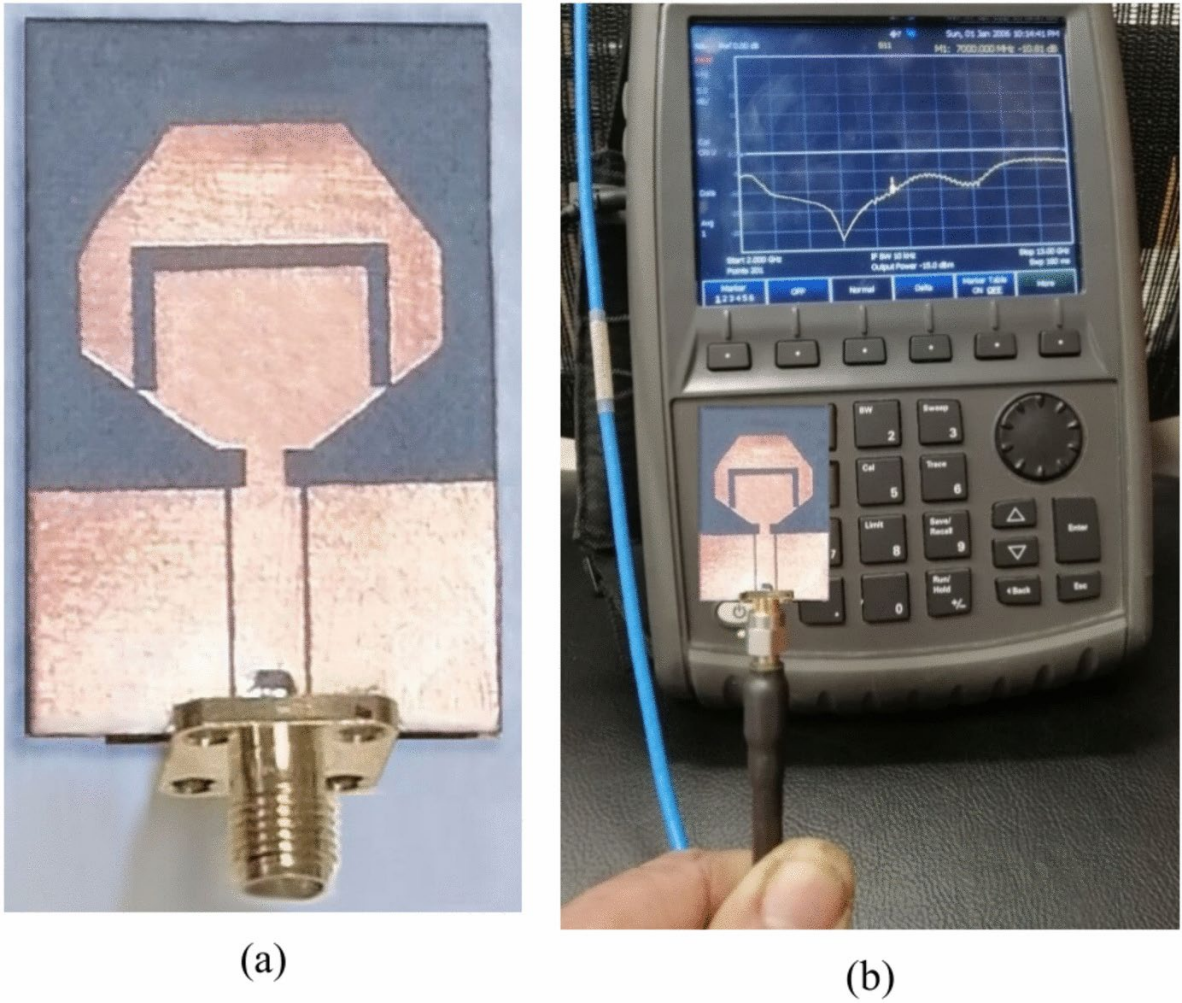
Experimental work. (**a**) Fabricated antenna. (**b**) The refelection coefficient is measured by the VNA over the frequency range $2-13\,\text{GHz}$.

**Fig. 7:**
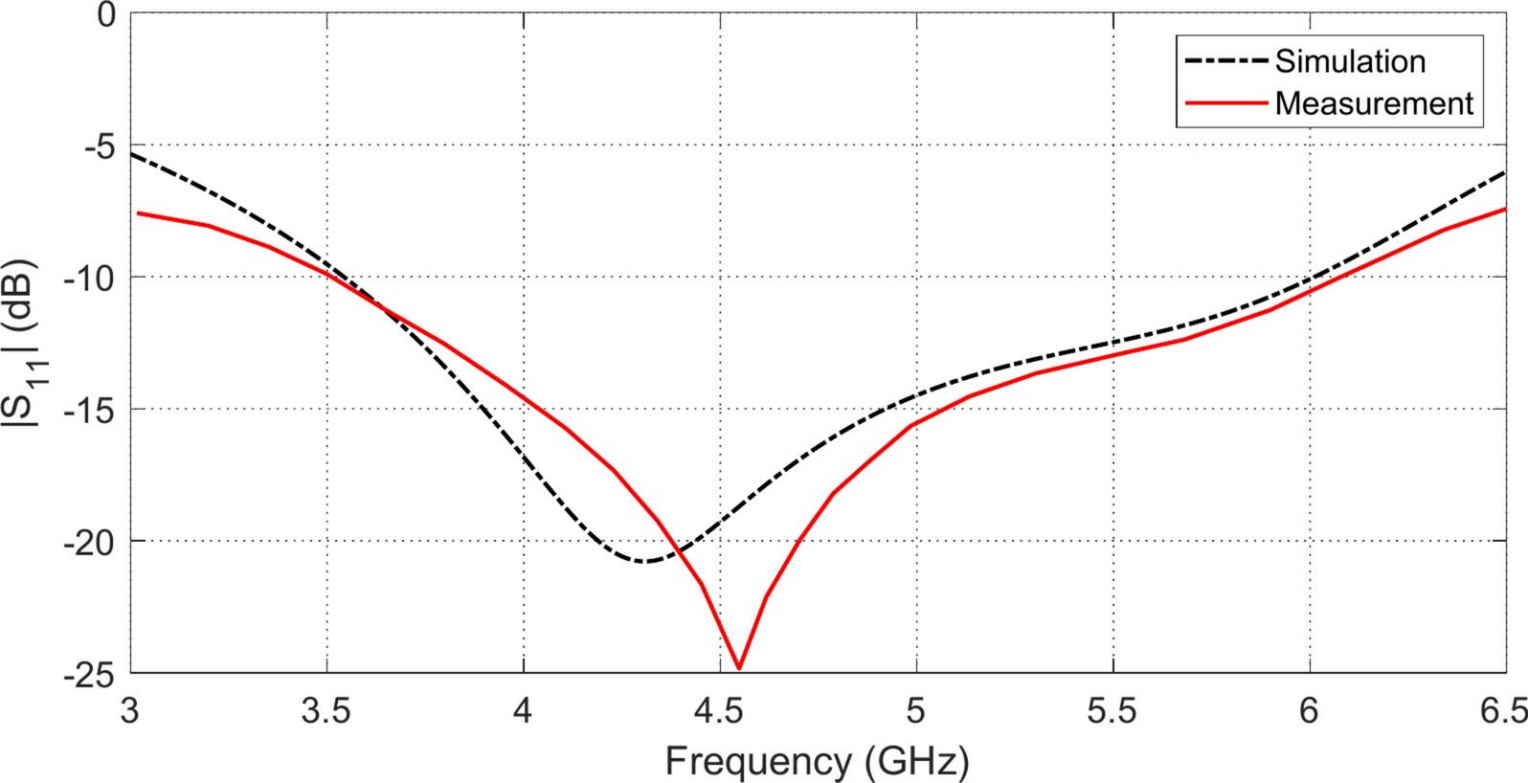
The frequency behavior of the reflection coefficient magnitude $\left|{S}_{11}\right|$ for the proposed wideband antenna as obtained by simulation and measurement.

For experimental evaluation of the proposed wideband antenna when placed in free space, the prototype shown in Fig. 6a in fabricated using the technology of lithography. The vector network analyzer (VNA) model Agilent N9918A is used for measuring the reflection coefficient ${S}_{11}$ of the antenna over the frequency range $2-13\,\text{ GHz}$ as shown in Fig. 6b. In Fig. 7, the measured frequency response of $\left|{S}_{11}\right|$ is compared to the corresponding results obtained by simulation showing good agreement with each other. The results show that the antenna impedance is matched to $50\Omega$ over the frequency band $3.5-6.0\,\text{GHz}$.

## Surface current distribution for the antenna

**Fig. 8:**
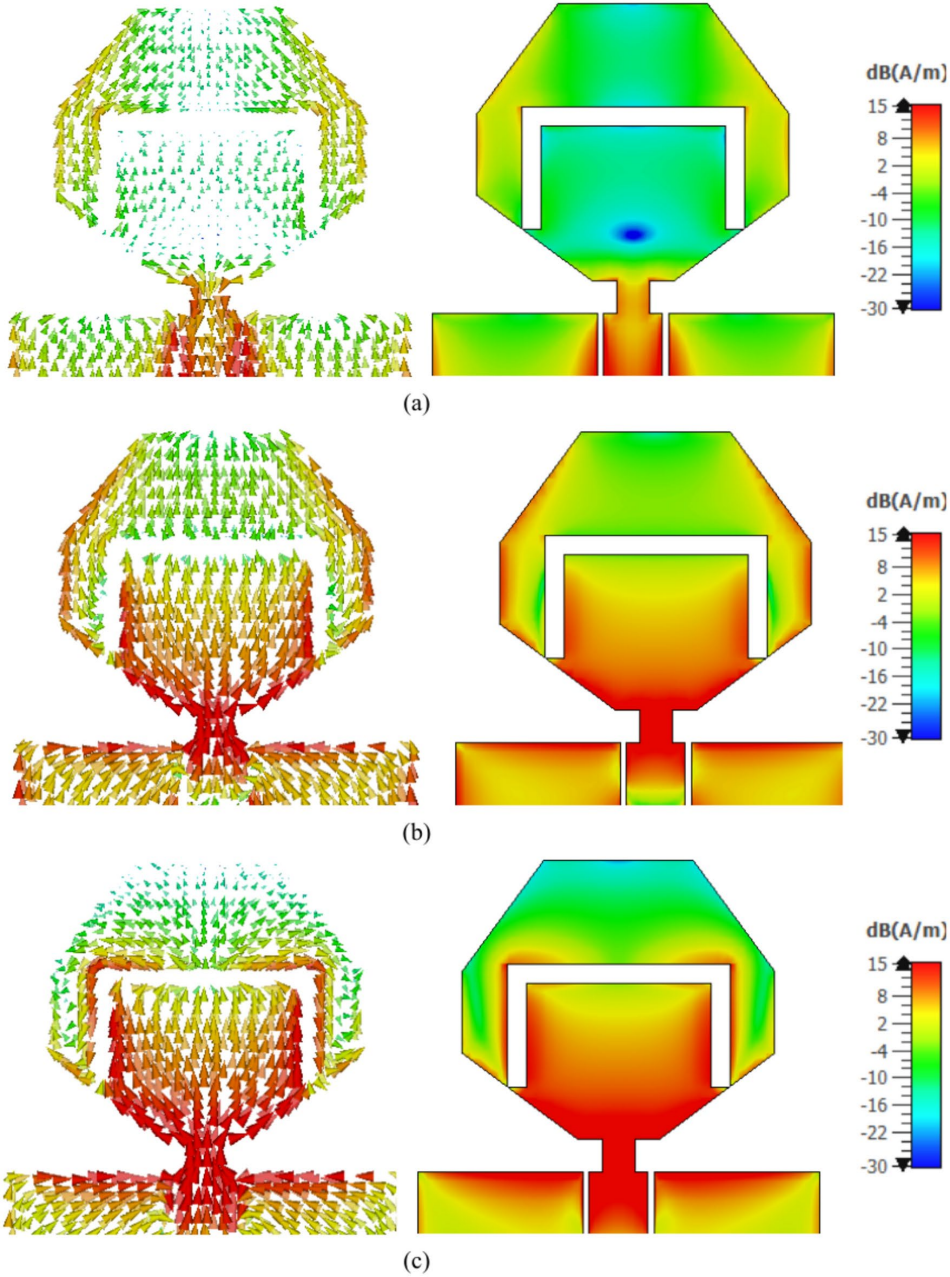
Surface current directions and density distributions at (**a**) 4 GHz, (**b**) 5 GHz, and (**c**) 6 GHz.

The radiation characteristics of the proposed planar antenna in free space can be explained in view of the current distribution on the antenna surface. The surface current distributions can give physical insight to interpret the radiated field associated by the different mechanisms of radiation at different frequencies over the wide frequency band of impedance matching. Figure 8 presents the surface current distributions on the surface of the radiating patch at 4, 5, and 6 GHz.

It is shown that the current density distribution is strongly dependent on the frequency. At 4 GHz, the current is mainly concentrated in the regions near the edges of the patch and the outer edges of the U-shaped slot. At this frequency, the surface current distribution exhibits 1st–order pattern. At 5 GHz, the surface current in concentrated in the lower half of the patch and in the regions near the patch edges and the inner edges of the U-shaped slot. The surface current distribution at 5 GHz still exhibits 1st–order pattern. At 6 GHz, the surface current is highly concentrated along the vertical edges and the corners of the U-shaped slot. It is clear that the surface current distribution exhibits, also, a 1st–order pattern. Thus, the current configuration on the patch surface maintains a 1st–order pattern over the entire frequency band. This means that the radiation patterns of the far field produced by the proposed antenna will have similar (omnidirectional) shapes over the wide frequency band of operation. Also, it is expected that the gain will increase with increasing the frequency due to the formation of the edge currents with higher density along the edges of the U-shaped slot with increasing the frequency.

## Radiation characteristics of the antenna in free space

### Radiation patterns

**Fig. 9:**
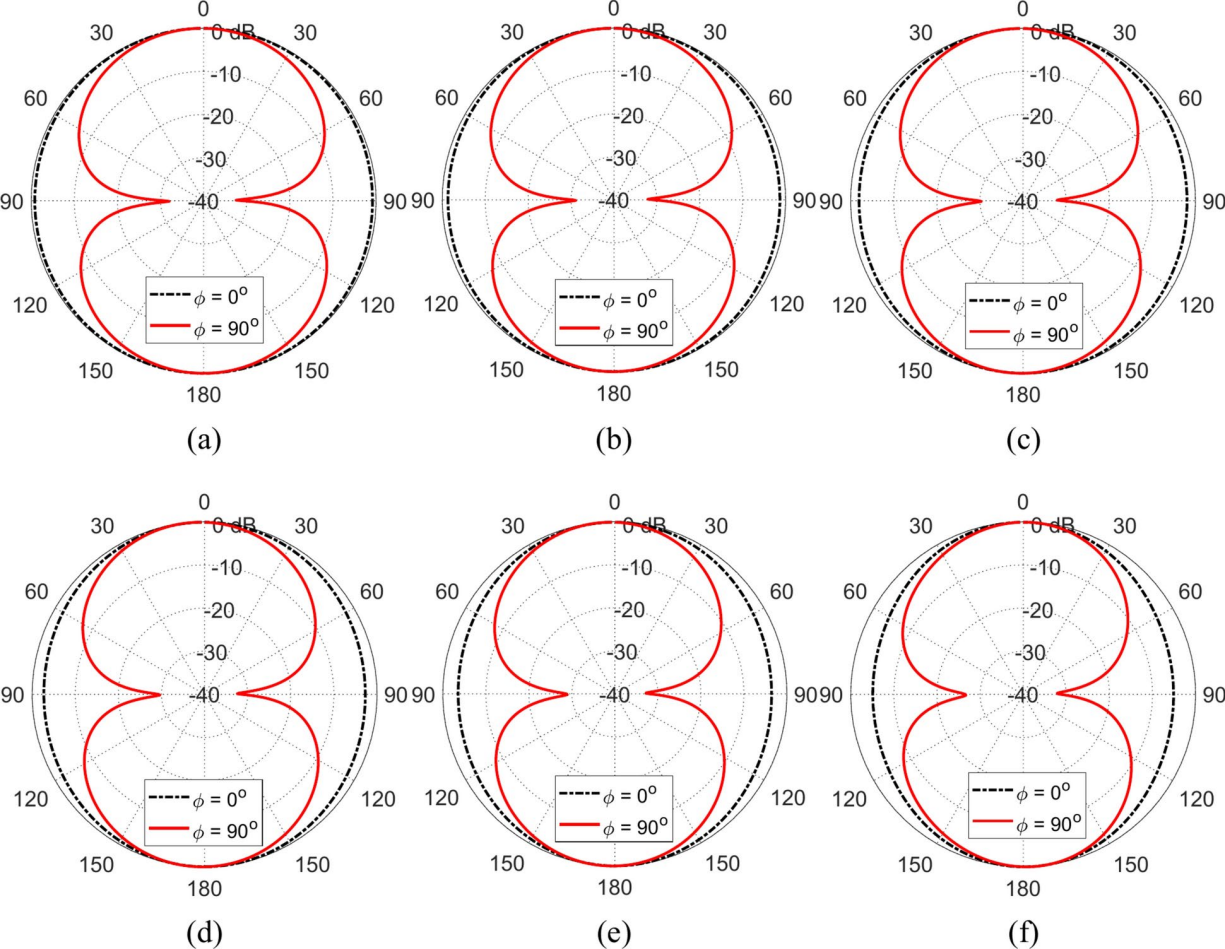
Radiation patterns of the proposed wideband antenna in the elevation planes $\phi ={0}^{^\circ }$ and $\phi ={90}^{^\circ }$ at (**a**) 3.5 GHz, (**b**) 4.0 GHz, (**c**) 4.5 GHz, (**d**) 5.0 GHz, (**e**) $5.5 \text{GHz}$, and (**f**) 6.0 GHz.

The radiation patterns produced at different frequencies over the operational frequency band when the proposed antenna is placed in free space are presented in Fig. 9. It is shown that, at all the frequencies, the radiated field has omnidirectional pattern with the figure-of-eight. It is also, clear that the width of the beam becomes slightly narrower with increasing the frequency.

### Frequency behavior of the antenna gain

**Fig. 10:**
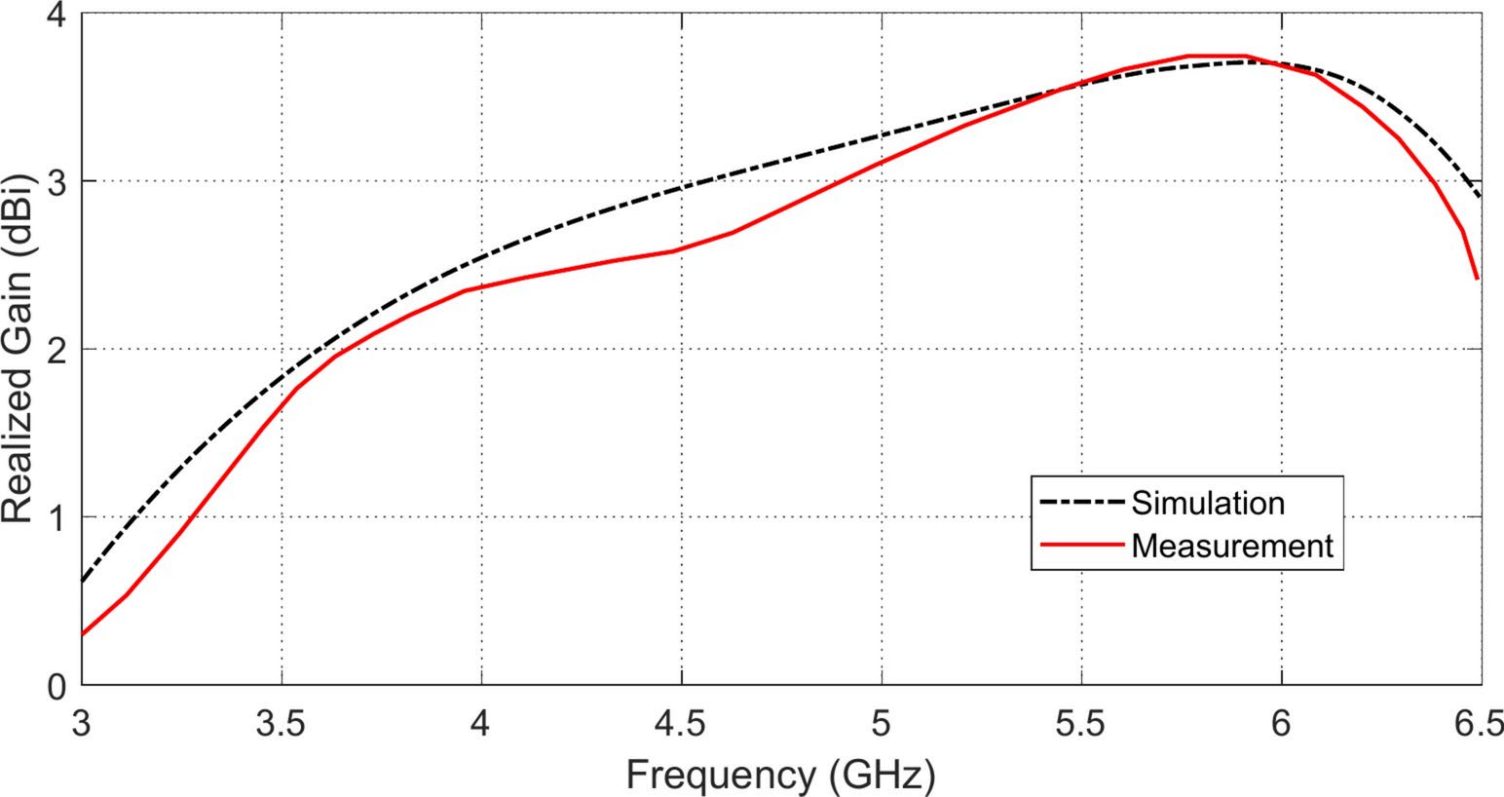
Frequency response of the realized gain of the proposed monopole patch antenna when placed in free space.

The frequency response of the realized antenna gain is presented in Fig. 10, where the experimental results show good agreement with the corresponding simulation results. It is shown that the antenna gain increases with the frequency, which comes in agreement with variation of the surface current distributions with increasing the frequency as previously explained in Section “Radiation characteristics of the antenna in free space”. Also, it is shown that, like an omnidirectional antenna, the gain ranges from 2.4 to 4.2 dBi with increasing the frequency over the operational frequency band of the antenna. It should be noted that the experimental setup and the method of measuring the realized gain are described later on (Section “Radiation patterns”).

### Antenna efficiency

**Fig. 11:**
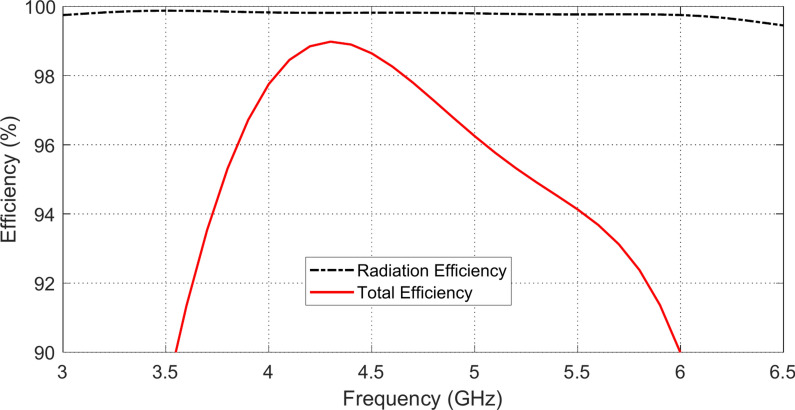
Frequency response of the radiation and total efficiencies of the proposed monopole patch antenna when placed in free space.

The frequency responses of the radiation and total efficiencies of the proposed wideband antenna when placed in free space are presented in Fig. 11. Owing to the single-side structure and the coplanar geometry of the antenna and CPW feeder, the radiation efficiency is almost 100% over the entire frequency band of operation. Also, owing to the properly matched impedance of the antenna, the total efficiency is maintained greater than 90% over the entire frequency band of impedance matching.

## AMC reflector design

This section provides a detailed description of the AMCS design process, beginning with the preliminary design equations, followed by the unit cell design, and concluding with the simulation that leads to the final AMCS design.

### Preliminary design equations of the AMCS

In the present context, the AMCS is materials designed to mimic the behavior of magnetically conducting surface in a way that can be used for enhancing the antenna performance regarding the radiation characteristics (gain and radiation efficiency). The design of AMCS typically involves the creation of structures that exhibit a frequency-dependent response that can be engineered to meet the goals of antenna design. While the design of AMCS can be complex and dependent on the particular use case, the basic preliminary design equations and presented to be customized to fulfill the design purposes.

The surface impedance ${Z}_{S}$ is one of the key parameters to describe an AMCS. The surface impedance for a perfect magnetic conductor (PMC) ideally has a value of zero. In the case of a structured AMCS, it can be approximated as

5 $${Z}_{S}=\frac{{V}_{S}}{{I}_{S}}$$

where ${V}_{S}$ and ${I}_{S}$ are the voltage and current at the surface of the AMC structure, respectively.

For AMCS, the goal is to design the surface impedance such that the material mimics a PMC in certain frequency bands. At these frequencies, the impedance should be enhanced (${Z}_{S}\to \infty$).

AMCs are often constructed using periodic structures such as patches or arrays of resonant elements (e.g., split-ring resonators). The resonant frequency ${f}_{0}$ of the AMC structure can be estimated using the dimensions of the unit cell and the material properties.

For an array of patches or resonators, the resonant frequency ${f}_{0}$ is often given by

6 $${f}_{0}=\frac{c}{{\lambda }_{0}}=\frac{c}{2d}$$

where $c$ is the speed of light in free space, ${\lambda }_{0}$ is the wavelength in free space, and $d$ is the characteristic dimension of the resonant structure (e.g., the side length of a square patch or the diameter of a circular patch, including spacing between resonators).

The reflection coefficient $R$ of the AMCS is important for determining how effectively the material reflects electromagnetic waves. The reflection coefficient is given by

7 $$R=\frac{{Z}_{S}-{Z}_{0}}{{Z}_{S}+{Z}_{0}}$$

where ${Z}_{0}$ is the free-space impedance (approximately $377\hspace{0.17em}\Omega$).

At the resonant frequency, for a well-designed AMCS, the surface impedance should theoretically be infinite (${Z}_{S}\to \infty$), leading to a unity reflection coefficient ($R\to \infty$) with zero-phase.

The effective permittivity (${\varepsilon }_{eff}$) and permeability (${\mu }_{eff}$) of an AMC are influenced by the geometry of the periodic elements. These can be approximated based on the structure of the unit cell (e.g., periodic patches, resonators, or loops). For an array of resonators, the effective permeability can be approximated as follows.

8 $${\mu }_{eff}=1-\frac{{\omega }^{2}{L}_{eff}}{{\omega }^{2}-{\omega }_{0}^{2}}$$

where ${L}_{eff}$ is the effective inductance of the resonators, and ${\omega }_{0}$ is the resonant angular frequency of the unit cell. The length of the unit cell is often chosen to be approximately one-quarter of the wavelength (L≈λ0/4) at the target frequency.

### Geometry of the unit cell of the proposed AMCS

**Fig. 12:**
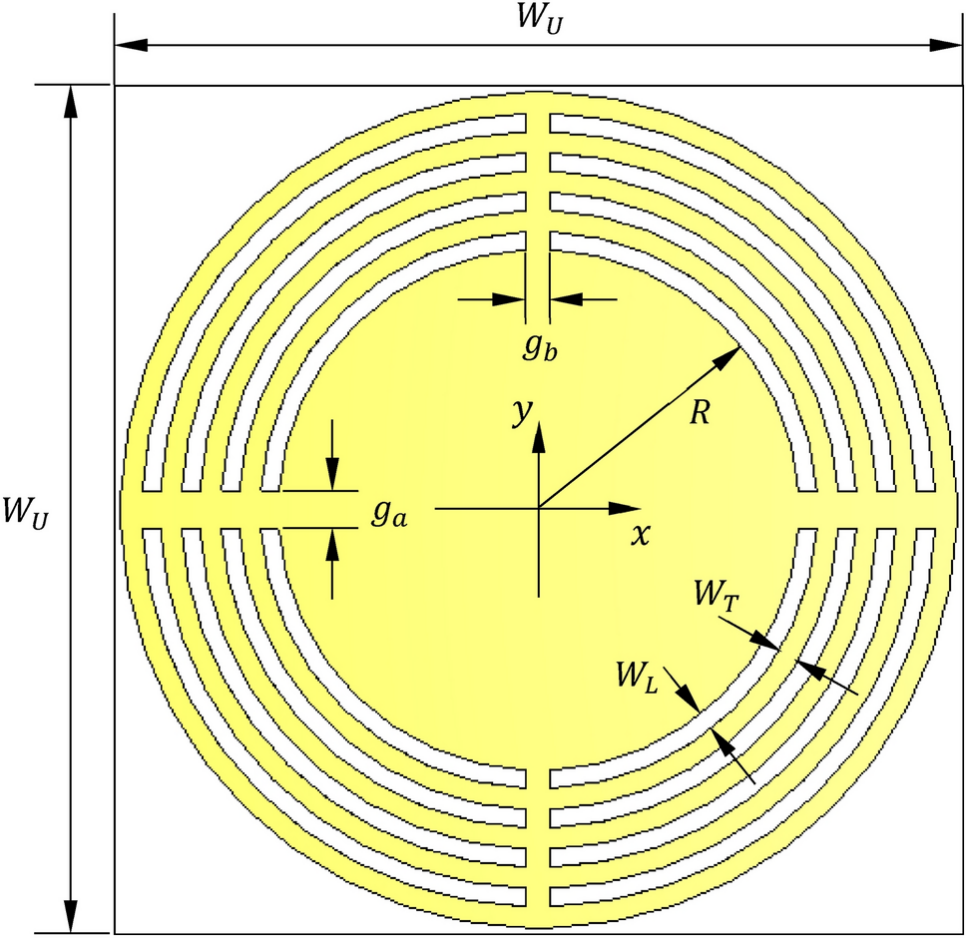
Geometry of the unit cell of the proposed AMCS.

**Fig. 13:**
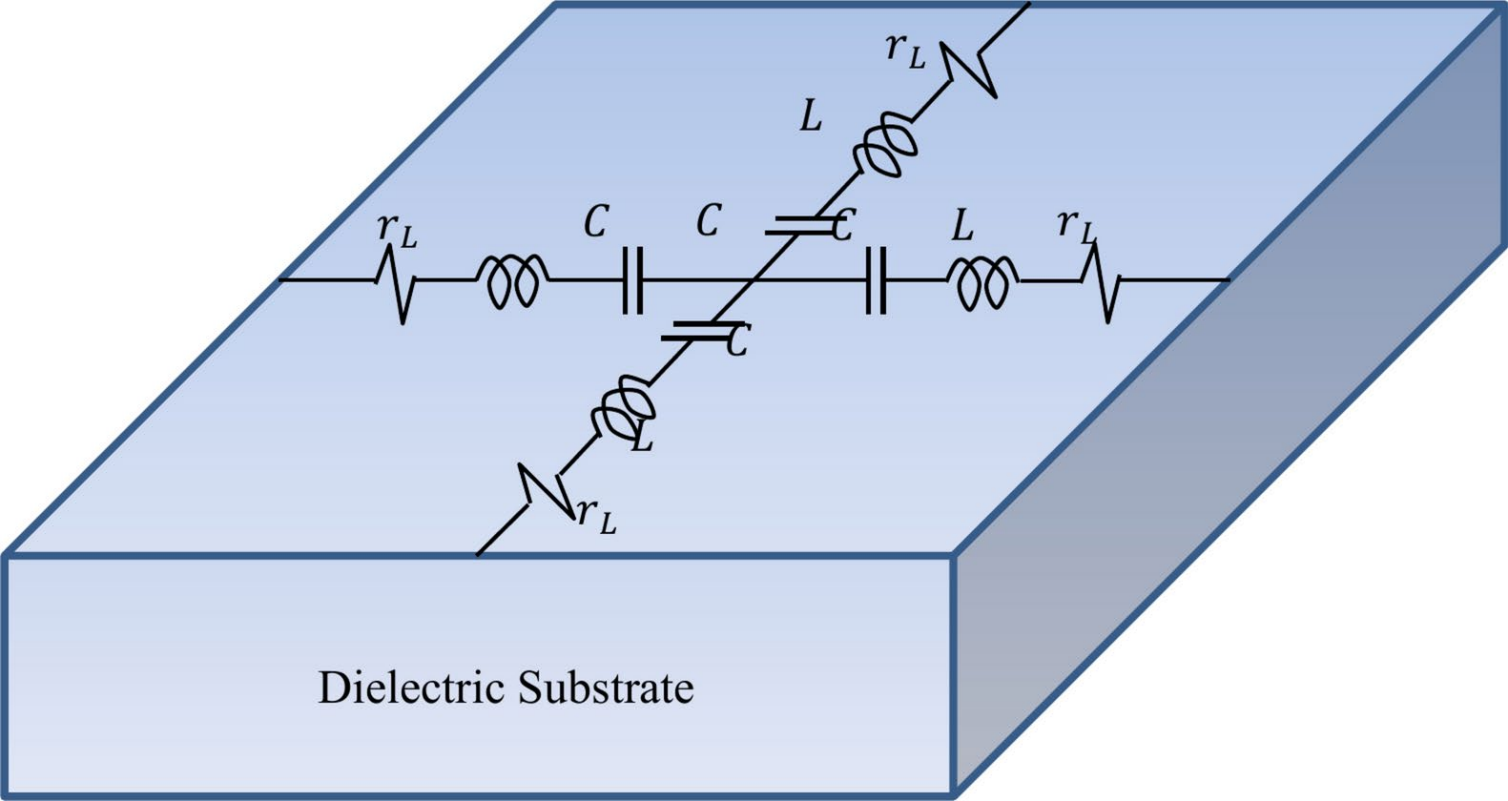
Unit cell equivalent circuit.

The proposed wideband antenna produces bidirectional radiation pattern with relatively low gain when placed in free space. Unidirectional radiation and, hence, gain enhancement can be achieved by placing the antenna at suitable height over a reflector to diminish back radiation and to enhance the forward radiation. This reflector is usually an electrically conducting surface (ECS) or AMC surface (AMCS). The ECS unifies the direction of radiation by reflecting the incident wave with $180^\circ$ phase shift, which requires the placement of the antenna at large enough height to avoid destructive interference between the incident and the reflected waves. The AMCS is a metasurface constructed as periodic structure to produce 0° reflection phase of the incident wave. Thus, the combined structure of the antenna and the AMCS reflector can have lower profile than that resulting in the case of employing ECS. The unit cell of the proposed AMCS has the geometry shown in Fig. 12. The Rogers’ RO4003C material is selected for the dielectric substrate that supports the periodic surface. This material has a dielectric constant ${\varepsilon }_{r}=3.38$, loss tangent $\text{tan}\delta =0.002$, and a thickness ${h}_{U}=1.524\text{ mm}$. The substrate is backed by solid metallic ground. The unit cell is composed of concentric metallic rings and slots with connecting strips as shown in Fig. 12. The unit cell of the proposed AMCS is square with dimensions 1$4\times 14\text{ mm}$. The dimensions of the AMC unit cell are listed in Table 2. For simulation, the unit cell of such periodic structure is modeled in the CST simulator and placed inside a bounding box as shown in Fig. 13. Periodic boundary conditions are applied on the side walls of the bounding box.

**Table 2:** Dimensions of the unit cell of the proposed AMCS.

Dimension	${W}_{U}$	$R$	${W}_{L}$	${W}_{T}$	${g}_{a}$	${g}_{b}$
Value (mm)	$14$	$4.3$	$0.3$	$0.35$	$0.6$	$0.4$

The equivalent circuit of the AMC unit cell can be represented as shown in Fig. 13. The interaction between each cell and its four neighboring cells is modeled as an RLC series connection^12^. The shunt components of the equivalent circuit are excluded due to the lack of a ground plane on the bottom surface of the unit cell. The series inductance, $L$, and capacitance, $C$, correspond to the components of the resonant circuit. The series resistance, ${r}_{L}$, accounts for the Ohmic losses in the periodic structure, which are primarily caused by dielectric losses.

Regarding Fig. 12, the separating distance between the neighboring cells, $G$, is given as

9 $$G= {W}_{U}-2\left[R+4\left({W}_{L}+{W}_{T}\right)\right]$$

The capacitance ($C$) between the neighboring cells is given as^17^.

10 $$C=\frac{{\varepsilon }_{r}{\varepsilon }_{0}}{\pi \left[R+4\left({W}_{L}+{W}_{T}\right)\right]}{\text{cosh}}^{-1}\left(\frac{{W}_{U}}{G}\right)$$

The inductance ($L$) between the neighboring cells is given as^17^.

11 $$L=\frac{\pi {\mu }_{r}{\mu }_{0}\left[R+4\left({W}_{L}+{W}_{T}\right)\right]}{{\text{cosh}}^{-1}\left(\frac{{W}_{U}}{G}\right)}$$

The resistance (${r}_{L}$) shown in Fig. 13 represents the dielectric material loss and, hence, it can be given as

12 $${r}_{L}=\frac{\text{tan}\delta }{2\pi fC}$$

where $\text{tan}\delta$ is the loss tangent.

At the resonant frequency of the AMC, the inductive and capacitive impedances effectively cancel each other out. Therefore, the resonant frequency (${f}_{0}$) of the AMC can be given as

13 $${f}_{0}=\frac{1}{2\pi \sqrt{LC}}$$

To get the AMCS resonant at a desired frequency, the last expression (13) can be used together with the expressions of $C$ and $L$ given by (10) and (11), respectively to calculate the corresponding dimensions of the unit cell.

**Fig. 14:**
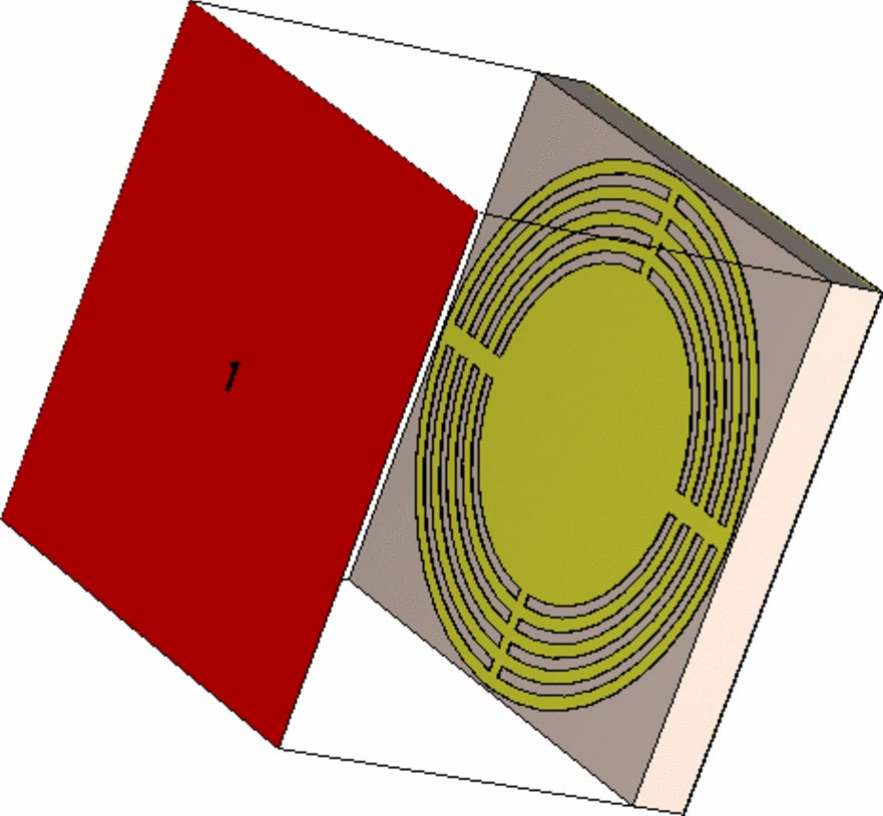
The excitation of the infinitely extending periodic struscture of the AMCS is achieved in the CST as one-port network as the substrate is backed by a solid metallic ground plane.

In the CST, the reflection characteristics of the AMCS are determined by applying a waveguide setup to mimic the infinite array of the AMCS. The setup consists of parallel E-plane and H-plane boundary conditions. The system is excited using a waveguide port placed on the top of the waveguide as shown in Fig. 14. To act as a base that enhances the gain of a low-gain antenna, the AMCS should be suitable for constructive interference with reflection coefficient of unity magnitude and phase shift lying in the range $-90^\circ$ to $+90^\circ$.

**Fig. 15:**
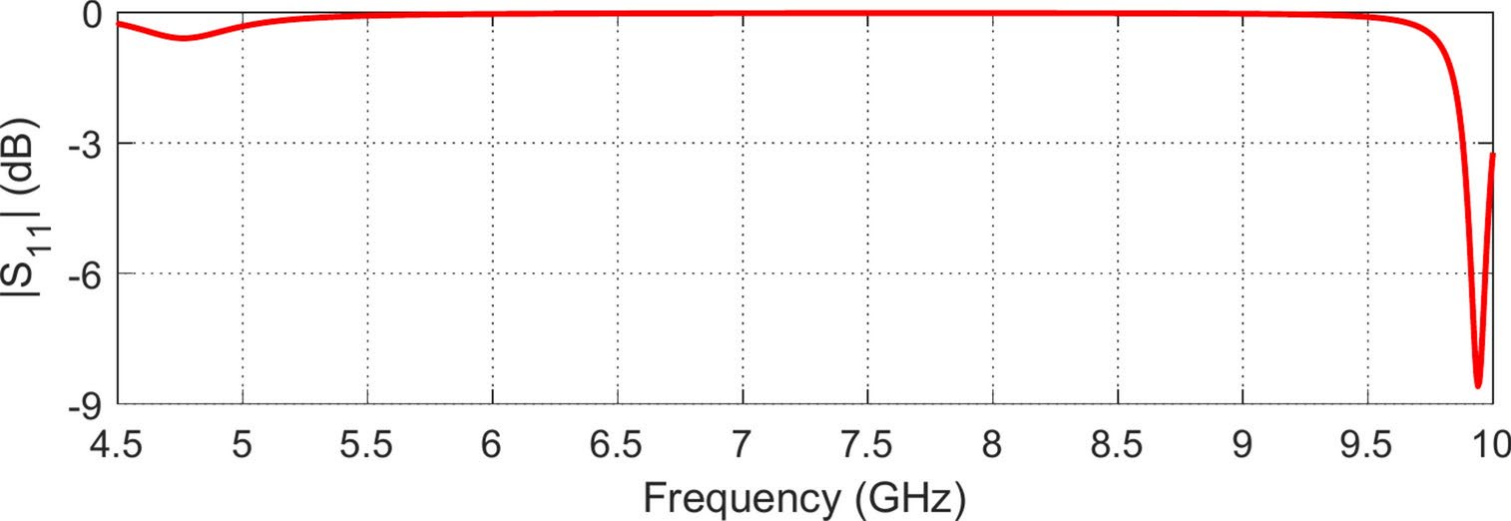
Reflection coefficient magnitude for the proposed AMC.

**Fig. 16:**
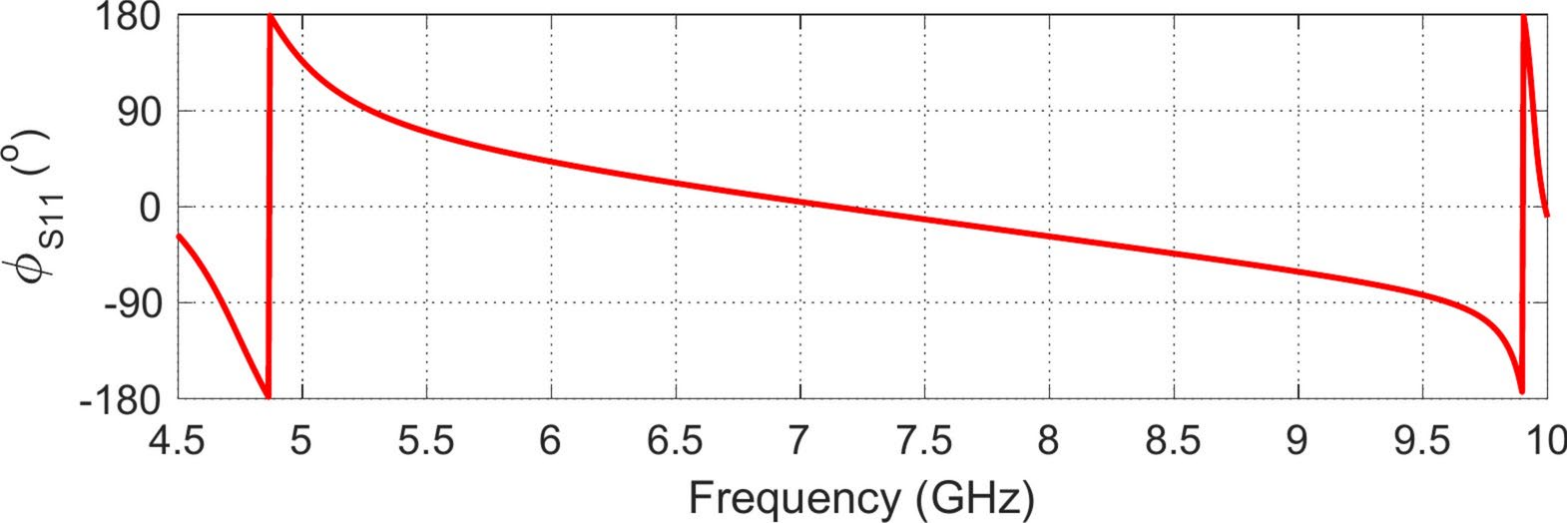
Variation of the reflection phase for the proposed AMCS.

In the simulation setup of the periodic planar structure, the system has a single waveguide port since the solid metallic ground on the bottom face of the dielectric substrate prevents the incident wave from being transmitted to the other side of the AMCS. Thus, only one scattering parameter (${S}_{11}$) exists whose magnitude and phase variations are shown in Figs. 15 and 16, respectively, over the frequency range 4.5–10 $\text{GHz}$. From the frequency behavior of the phase of the reflection coefficient, the AMCS has resonance at $7.13\text{ GHz}$. The bandwidth is defined as the range of frequencies over which the phase is maintained within the limits $[-90^\circ , +90^\circ$] around the resonant frequency. Thus, the frequency band of the proposed AMCS is 5.27–9.61 GHz.

## Performance of the monopole patch antenna when placed over AMCS

**Fig. 17:**
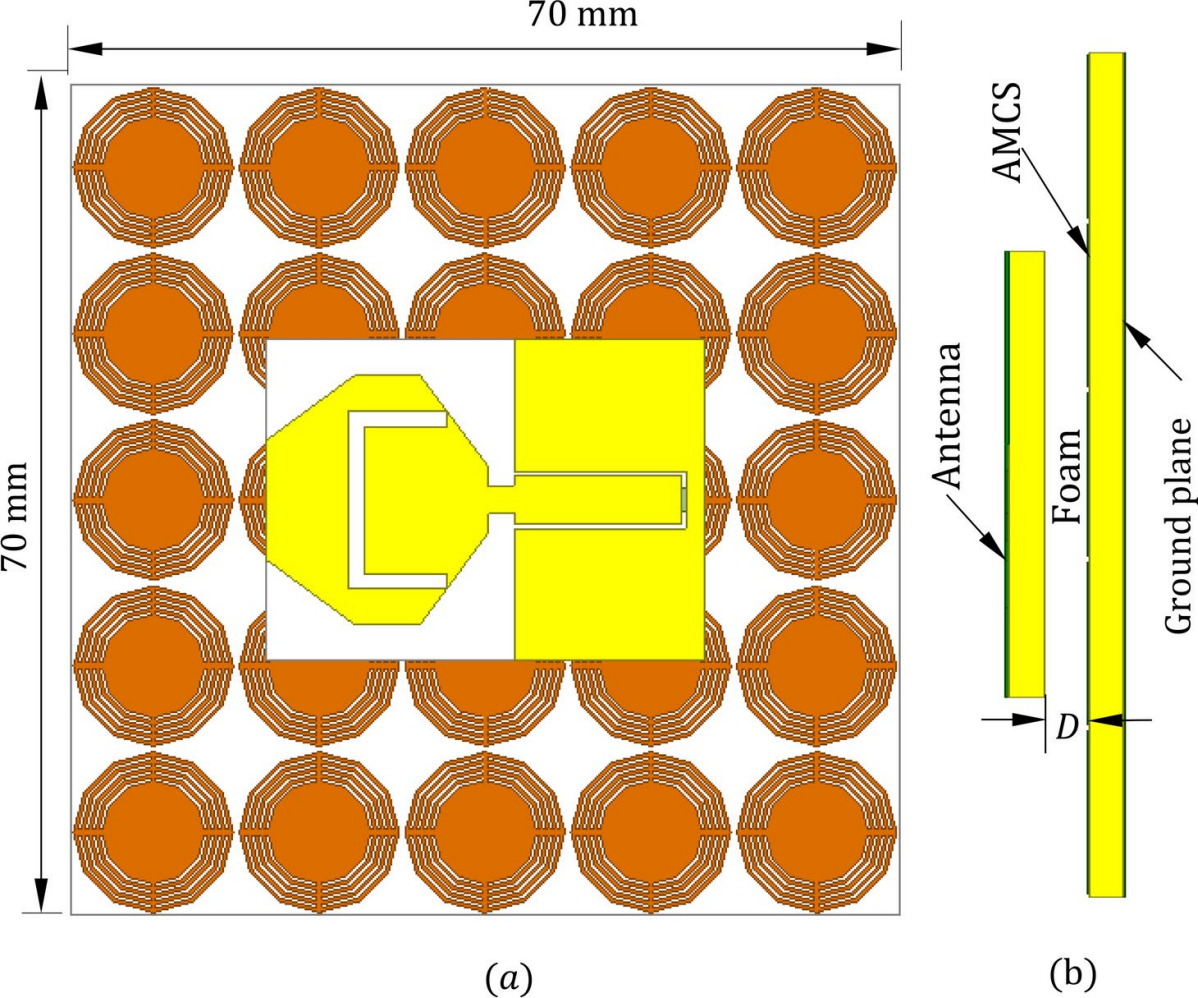
The monopole octagonal patch antenna placed at a height $D=5 \text{mm}$ above an AMCS of $5\times 5$ cells. (**a**) Top view. (**b**) Side view.

The radiation performance of the proposed antenna can be improved by placing it at a suitable height above an AMCS of appropriate dimensions. The performance improvement involves the production of unidirectional radiation and gain enhancement. Figure 17 shows the monopole octagonal patch antenna placed over a finite AMCS of 5 × 5 cells and dimensions $70\text{ mm }\times 70\text{ mm}$. The metallic patches of AMCS cells are printed on the top layer of a substrate of type Rogers’ RO4003C as described in Section “Experimental work of the antenna in free space”. A foam block separator of thickness $D$ is placed between the antenna and the AMCS. The foam material resembles the free space as it has dielectric constant ${({\varepsilon }_{r}}_{Foam}\approx 1.0$) and conductivity ${\sigma }_{Foam}\approx 1.0$.

The placement of the AMCS below the antenna has significant effects on the impedance matching and its bandwidth, radiation pattern, gain, and antenna efficiency. Definitely, the height, $D$, of the antenna over the AMCS has major impact on the antenna performance. The following subsections are dedicated to present quantitative analysis of such performance metrics.

### Effect of the AMCS on the impedance matching

**Fig. 18:**
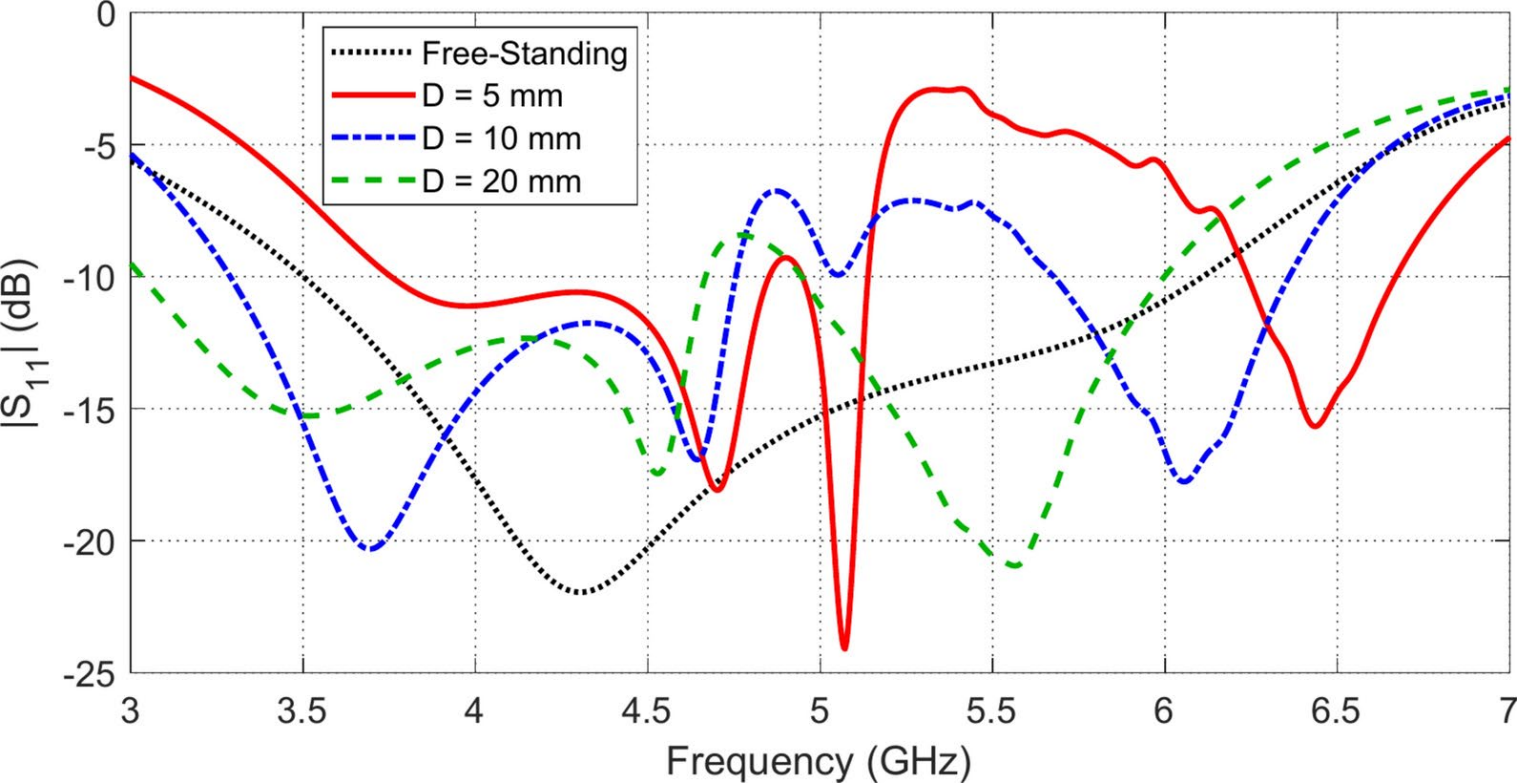
Frequency dependence of $\left|{S}_{11}\right|$ when the antenna is placed at different heights over an AMCS of $5\times 5$ cells and dimensions $70 \text{mm}\times 70 \text{mm}$, compared to $\left|{S}_{11}\right|$ of the antenna without AMCS.

The placement of the AMCS below the antenna significantly affects the frequency band over which the antenna impedance is matched to $50\Omega$ as shown in Fig. 18. For example, the placement of the antenna at a height $D=5 \text{mm}$ over the AMCS divides the frequency band of impedance matching into two bands; the lower band is ($3.7-5.2\text{ GHz}$) and the upper band is ($6.2-6.7\text{ GHz}$). It is shown that the bandwidth of impedance matching is increased with increasing the height $D$.

### Gain improvement by the AMCS

**Fig. 19:**
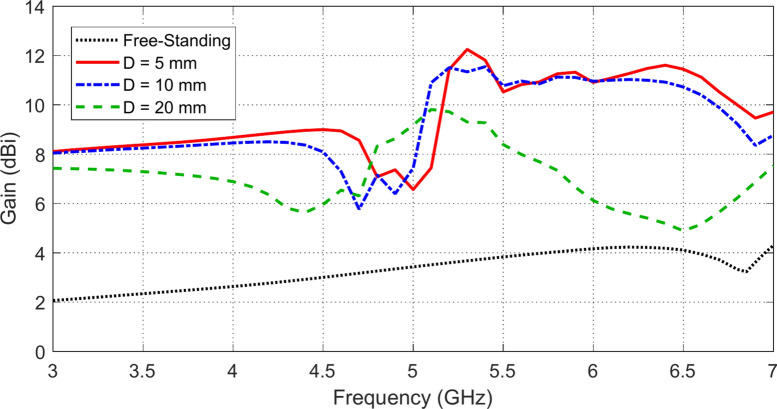
Frequency dependence of the maximum gain when the antenna is placed at different heights over an AMCS of $5\times 5$ cells and dimensions $70 \text{mm}\times 70 \text{mm}$, compared to the gain obtained by the antenna without AMCS.

The placement of the AMCS below the antenna enhances the antenna gain over the entire frequency band as shown in Fig. 19. It is noticed the gain increased with reducing the antenna height, $D$, above the AMCS. For example, the placement of the antenna at a height $D=5 \text{mm}$ over the AMCS enhances the antenna gain to 8.7 dBi and 11.5 dBi over the lower and upper frequency bands of impedance matching ($3.7-5.2\text{ GHz}$, and $6.2-6.7\text{ GHz}$), respectively.

### Radiation patterns of the antenna over the AMCS

**Fig. 20:**
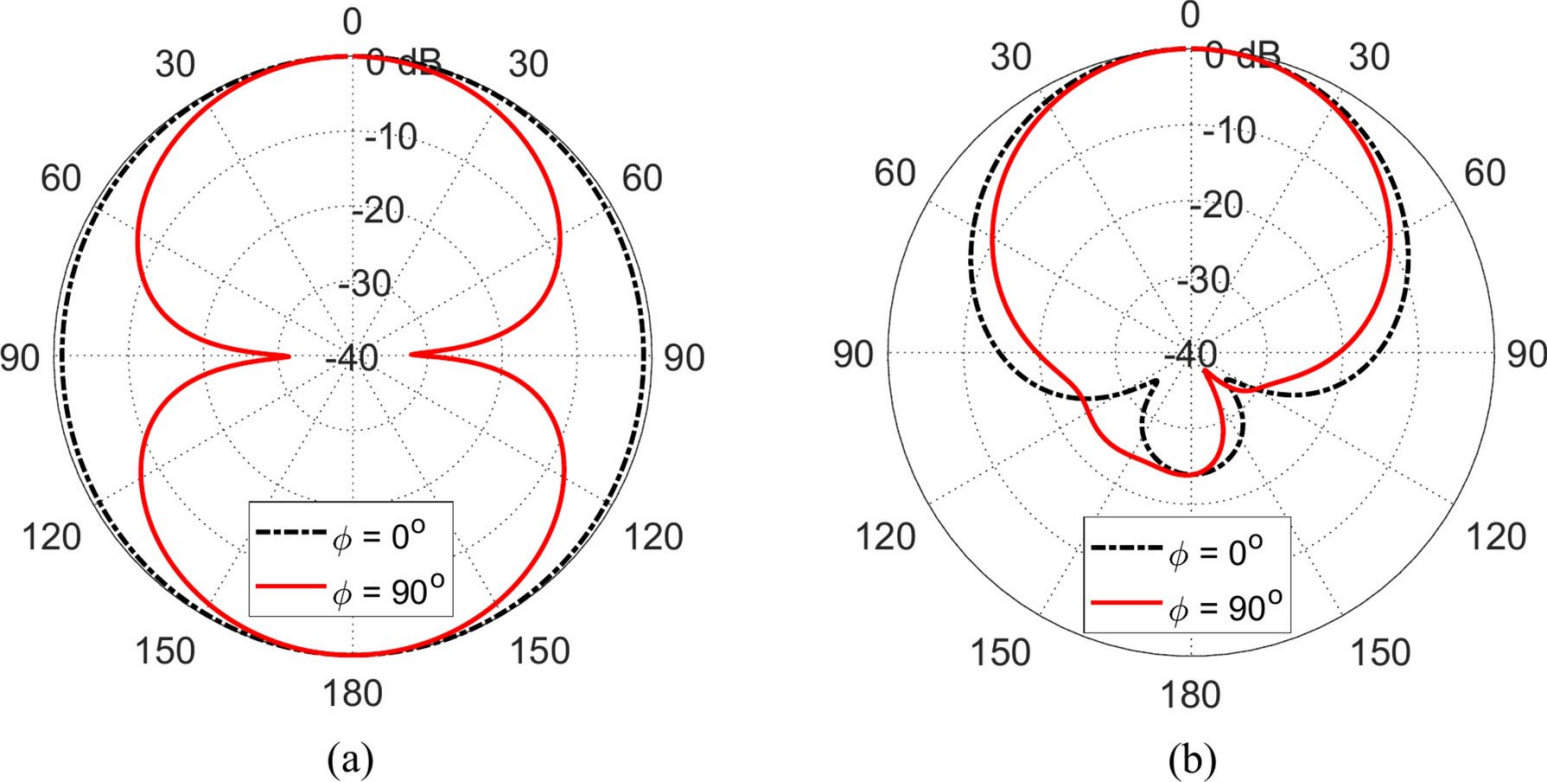
Radiation patterns obtained by simulation at $4\text{ GHz}$ when the proposed monopole patch antenna is placed (**a**) in free space, (**b**) at a height $D=5\text{ mm}$ over an AMCS of $5\times 5$ cells and dimensions $14 \text{cm}\times 14 \text{cm}$.

**Fig. 21:**
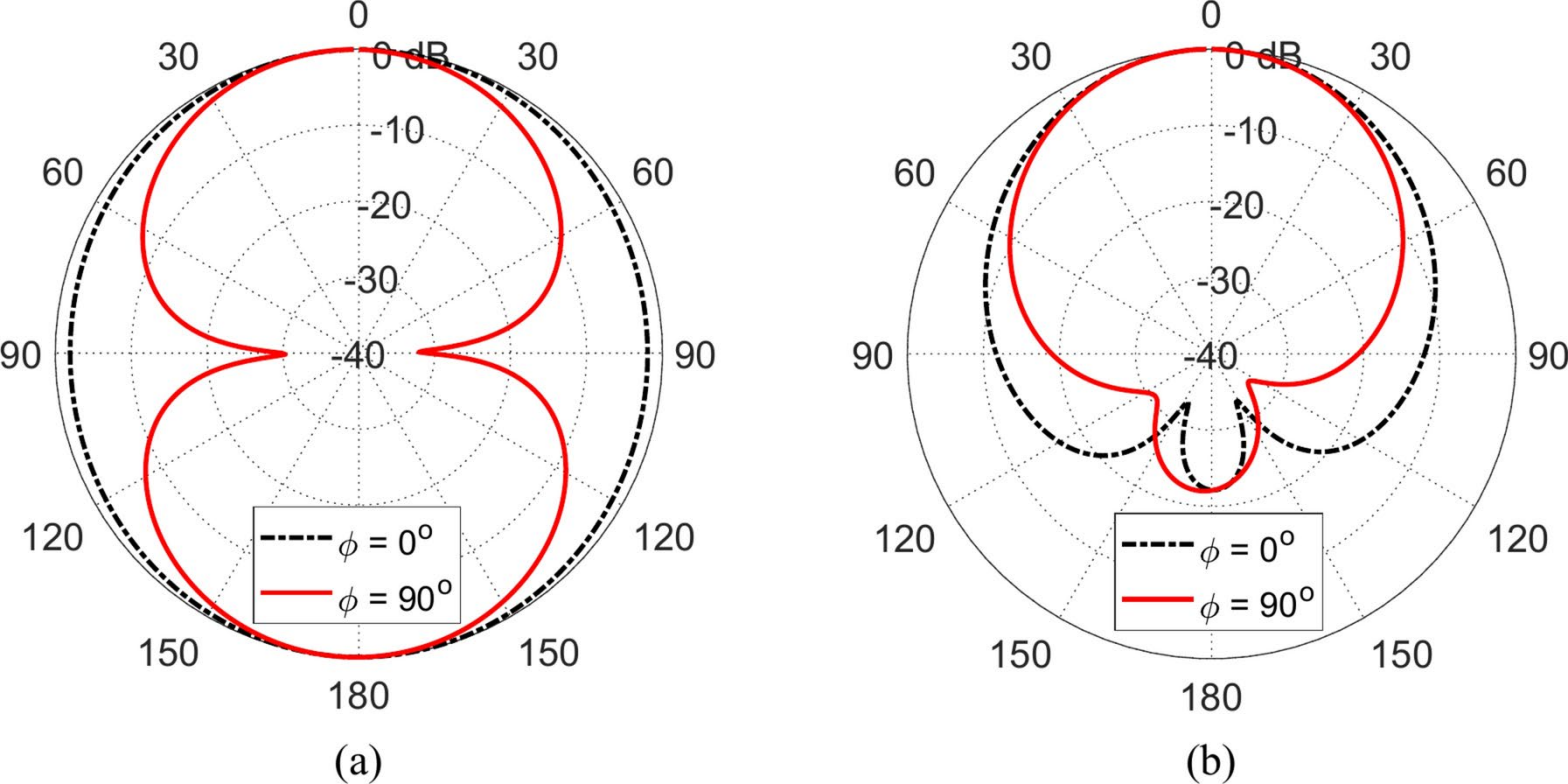
Radiation patterns obtained by simulation at $5\text{ GHz}$ when the proposed monopole patch antenna is placed (**a**) in free space, (**b**) at a height of $D=5\text{ mm}$ over an AMCS of $5\times 5$ cells and dimensions $14 \text{cm}\times 14 \text{cm}$.

When the propesed wideband monopole patch antenna is placed in free space it produces omnidirectional radiation with the figure-of-eight patterns presented in Figs. 20a and 21a at 4 and 5 GHz, respectively. When placed at a height $D=5\text{ mm}$ over an AMCS of $5\times 5$ cells and dimensions $14 \text{cm}\times 14 \text{cm}$, the new structure produces unidirectional radiation patterns as shown in Figs. 20b, and 21b at 4 and 5 GHz, respectively. Due to instructive interference between the incident wave and that reflected by the AMCS, the forward radiation is enhanced whereas the back radiation is diminished leading to unidirectional radiation and gain enhancement.

## Experimental investigation of the effects of the AMCS on the antenna performance

The effects of the AMCS on the impedance matching bandwidth and the realized gain are investigated experimentally.

### Fabrication of the AMCS

**Fig. 22:**
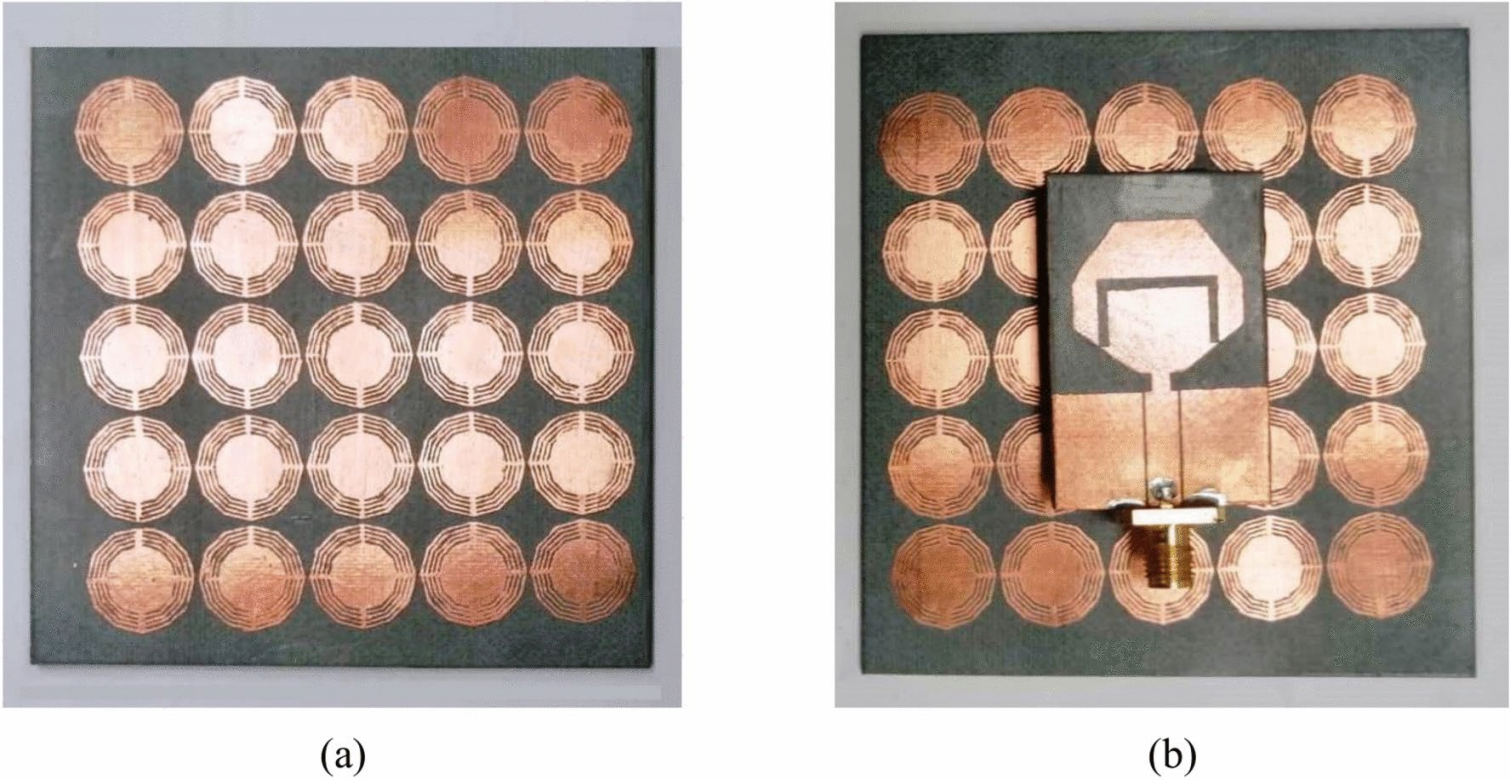
The proposed AMCS is composed of $5\times 5$ cells and has dimensions of $70 \text{mm}\times 70 \text{mm}$. (**a**) Fabricated AMCS. (**b**) The fabricated antenna is placed over the fabricated AMCS.

The AMCS presented in Fig. 17 is fabricated by lithography on a dielectric substrate of type Rogers’ RO4003C of thickness $h=1.52\text{ mm}$. The fabricated AMCS is presented in Fig. 22a. The fabricated antenna is placed at a height $D=5\text{ mm}$ above the AMCS for experimental measurements as shown in Fig. 22b.

### Measurement of the impedance matching bandwidth

**Fig. 23:**
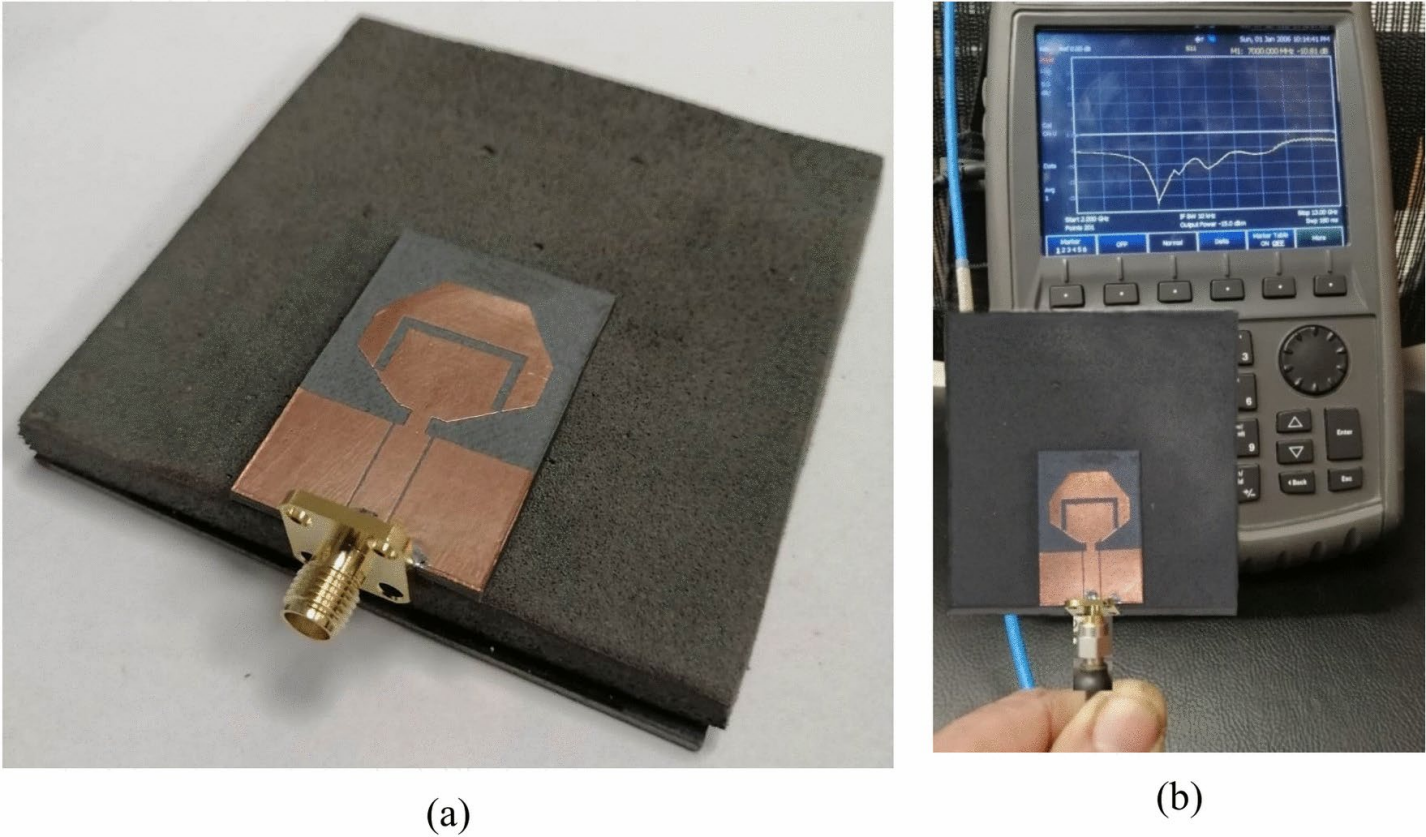
Experimental work for measuring the impedance matching bandwidth of the antenna when placed over the proposed AMCS. (**a**) Fabricated antenna placed over the AMCS using a block of foam. (**b**) Measurement of ${S}_{11}$ over the frequency range $2-13\text{ GHz}$.

To get the antenna structure over the AMCS robust during measurement, a block of foam (${{\varepsilon }_{r}}_{Foam}\approx 1,\text{tan}{\delta }_{Foam}\approx 0$) of thickness $D=5\text{ mm}$ is used as a supportive structure as shown in Fig. 23a. The reflection coefficient, ${S}_{11}$, of the antenna when placed is placed above the AMCS using the VNA over the frequency range $2-13\text{ GHz}$ as shown in Fig. 23b

**Fig. 24:**
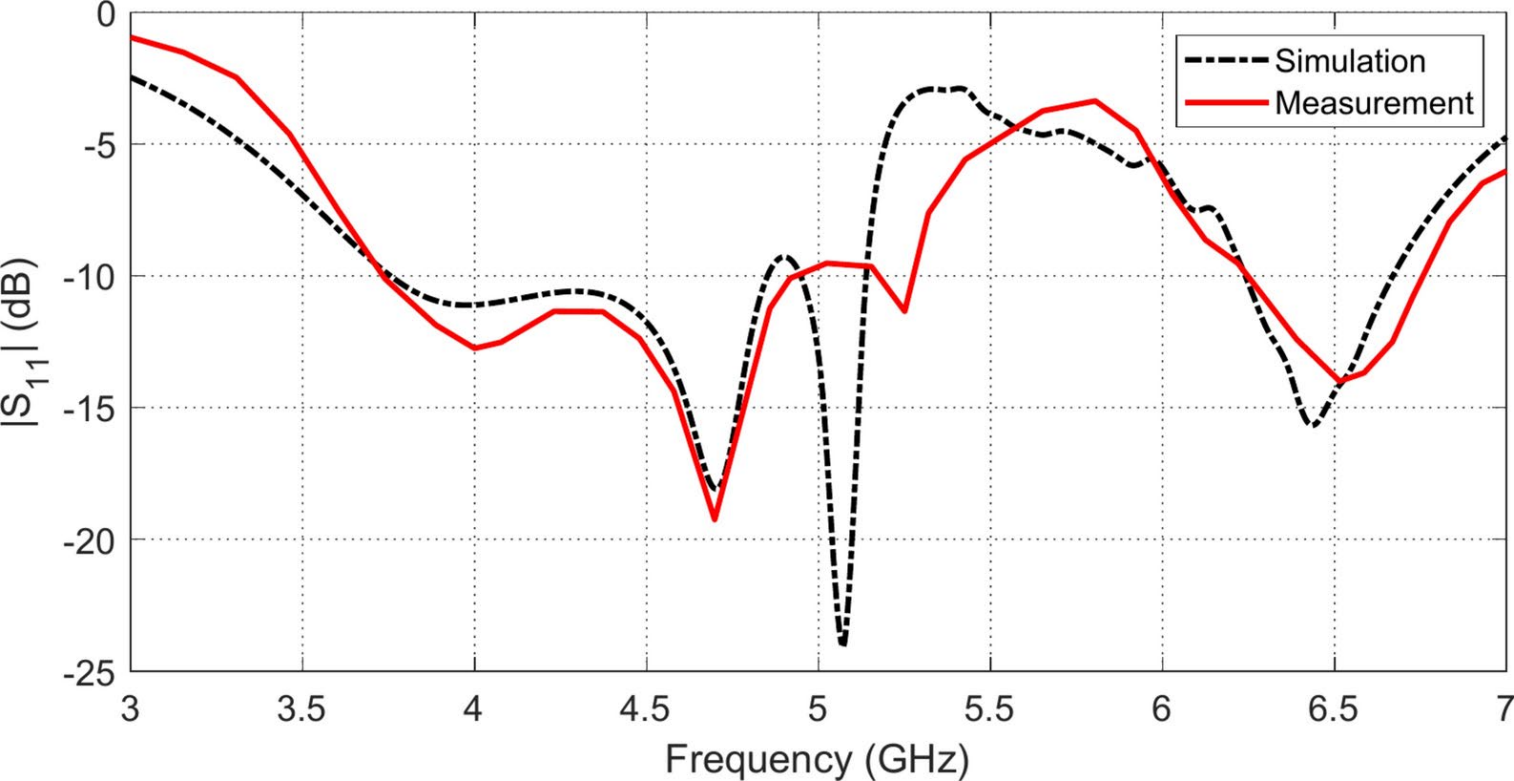
Comparison between the frequency dependence of $\left|{S}_{11}\right|$ obtained by simulation and measurement for the proposed antenna when placed at a height $D=5 \text{mm}$ above an AMCS of $5\times 5$ cells.

The frequency dependencies of $\left|{S}_{11}\right|$ as obtained by simulation and measurement for the proposed antenna when placed at a height $D=5 \text{mm}$ above an AMCS of $5\times 5$ cells, are presented in Fig. 24. It is shown that the results of simulation agree well with that of measurement. As shown in Fig. 23, the placement of the antenna above the AMCS results in changing the frequency band of impedance matching (of the free-standing antenna shown in Fig. 7). The composite radiating structure has two frequency bands of impedance matching; the lower frequency band is $3.7-5.2 \text{GHz}$ and the upper frequency band is $6.2-6.7 \text{GHz}$. This is attributed to the strong coupling between the antenna and the cells of the AMCS due to the small separation between them ($D=5 \text{mm}$).

### Measurement of the gain

**Fig. 25:**
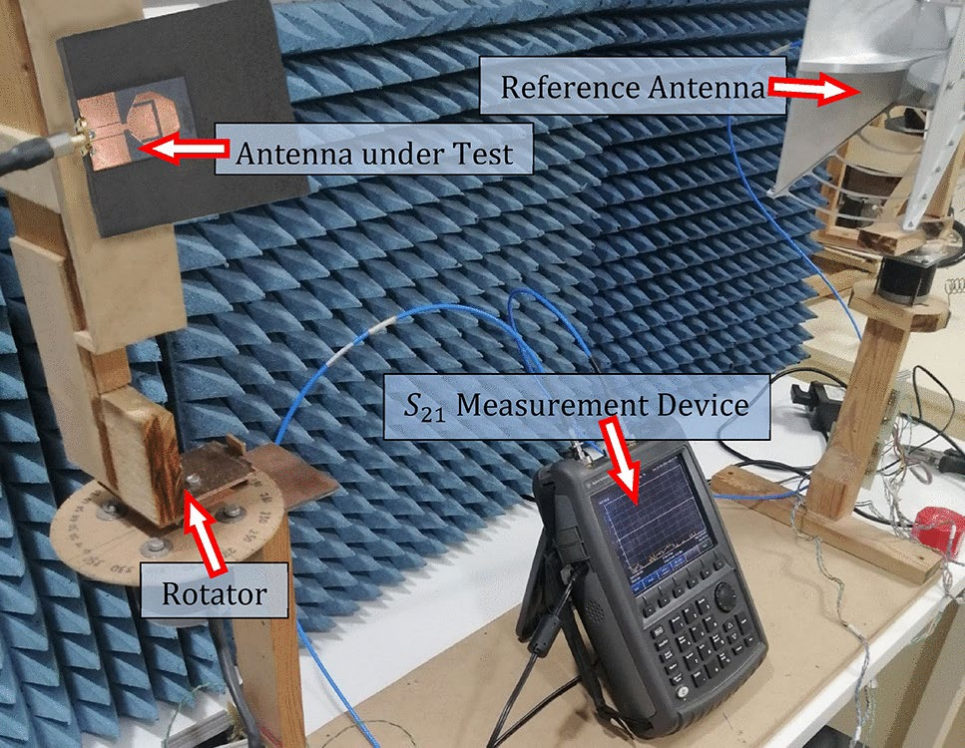
Measurement of the gain and radiation pattern.

The experimental setup presented in Fig. 25 is used for measuring the gain and radiation pattern of the radiating structure composed of the antenna over the AMCS. The antenna under test (AUT) is connected to port 1 of the VNA whereas the reference antenna (RA) is connected to port 2. The measured S-parameter ${S}_{21}$ between the AUT and the RA (during the rotation of the AUT) is used to measure the far field quantities such as the realized gain, radiation pattern and antenna efficiency. The method of measurement is described in detail in^13^ and^14^.

**Fig. 26:**
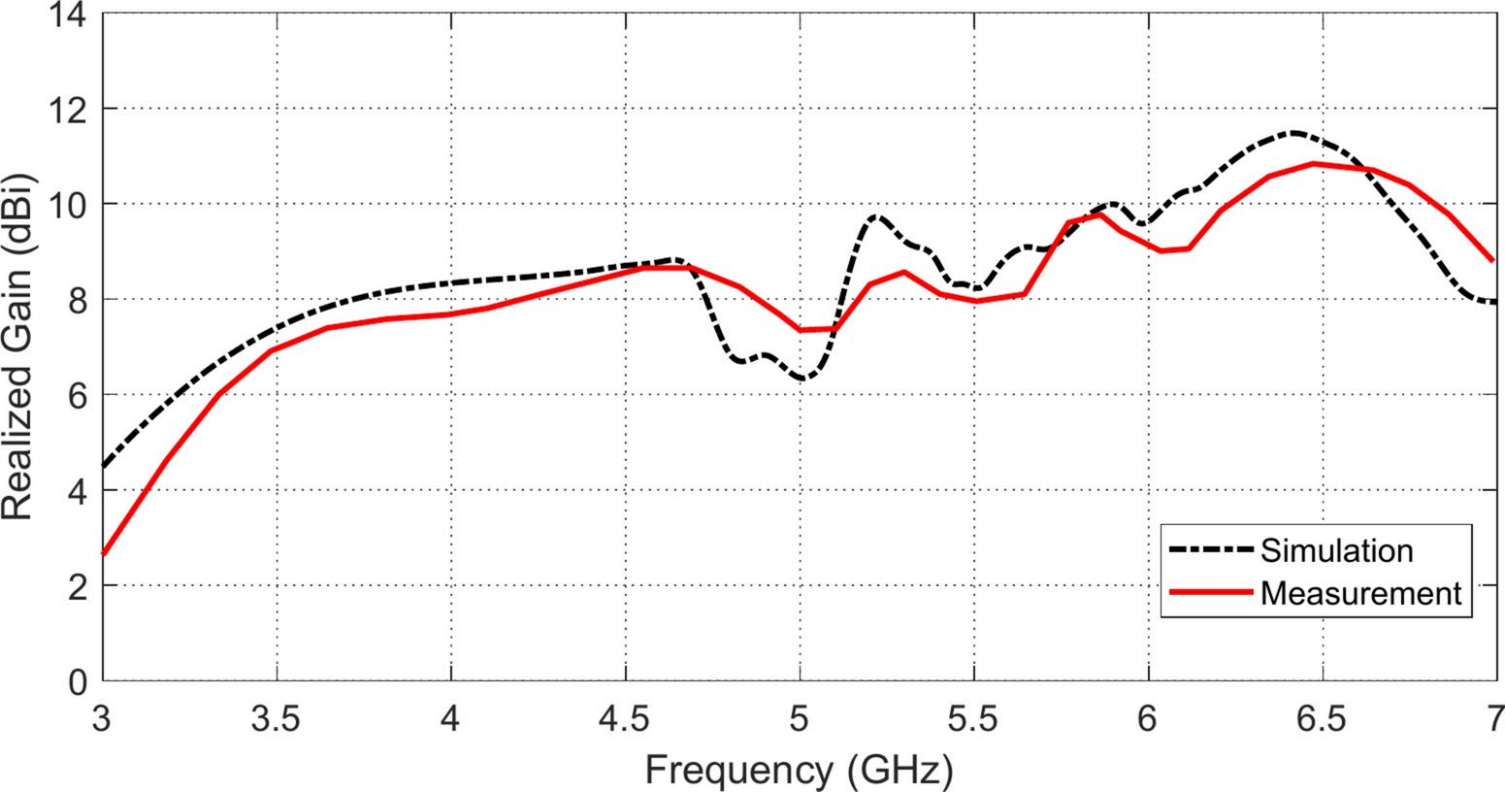
Comparison between the frequency dependence of the realized gain obtained by simulation and measurement for the proposed antenna when placed at a height $D=5 \text{mm}$ above an AMCS of $5\times 5$ cells.

The frequency dependencies of the realized gain as obtained by simulation and measurement for the proposed antenna when placed at a height $D=5 \text{mm}$ above an AMCS of $5\times 5$ cells are presented in Fig. 26. It is shown that the measured realized gain agrees well with that obtained by simulation. By comparing the between Figs. 26 and 10, it becomes clear that the placement of the proposed AMCS at a distance $D=5 \text{mm}$ below the planar monopole antenna leads to considerable enhancement of the realized gain relative to that of the free-standing antenna.

## Comparing the effects of the ECS and AMCS on the antenna performance

In this section, the performance of the proposed omnidirectional antenna when placed at a given height, $D$, over a square AMCS of dimensions $70 \text{mm}\times 70\text{ mm}$ and constructed of $5\times 5$ cells are compared to the performance of the same antenna when placed at the same height over a square ECS of the same dimensions as the AMCS. The ECS acts a reflector with $180^\circ$-phase reflection whereas the AMCS acts as a reflector with $0^\circ$-phase reflection. Both types of reflecting surface are able to produce unidirectional radiation pattern with enhanced gain when the antenna is placed at the suitable height over the reflecting surface. In the following, some of the results are presented for investigating the effect of employing both types of reflectors on the antenna performance regarding the impedance matching frequency band, the realized gain in the forward direction, and the radiation and total efficiencies of the radiating structure.

### Impedance matching and gain

**Fig. 27:**
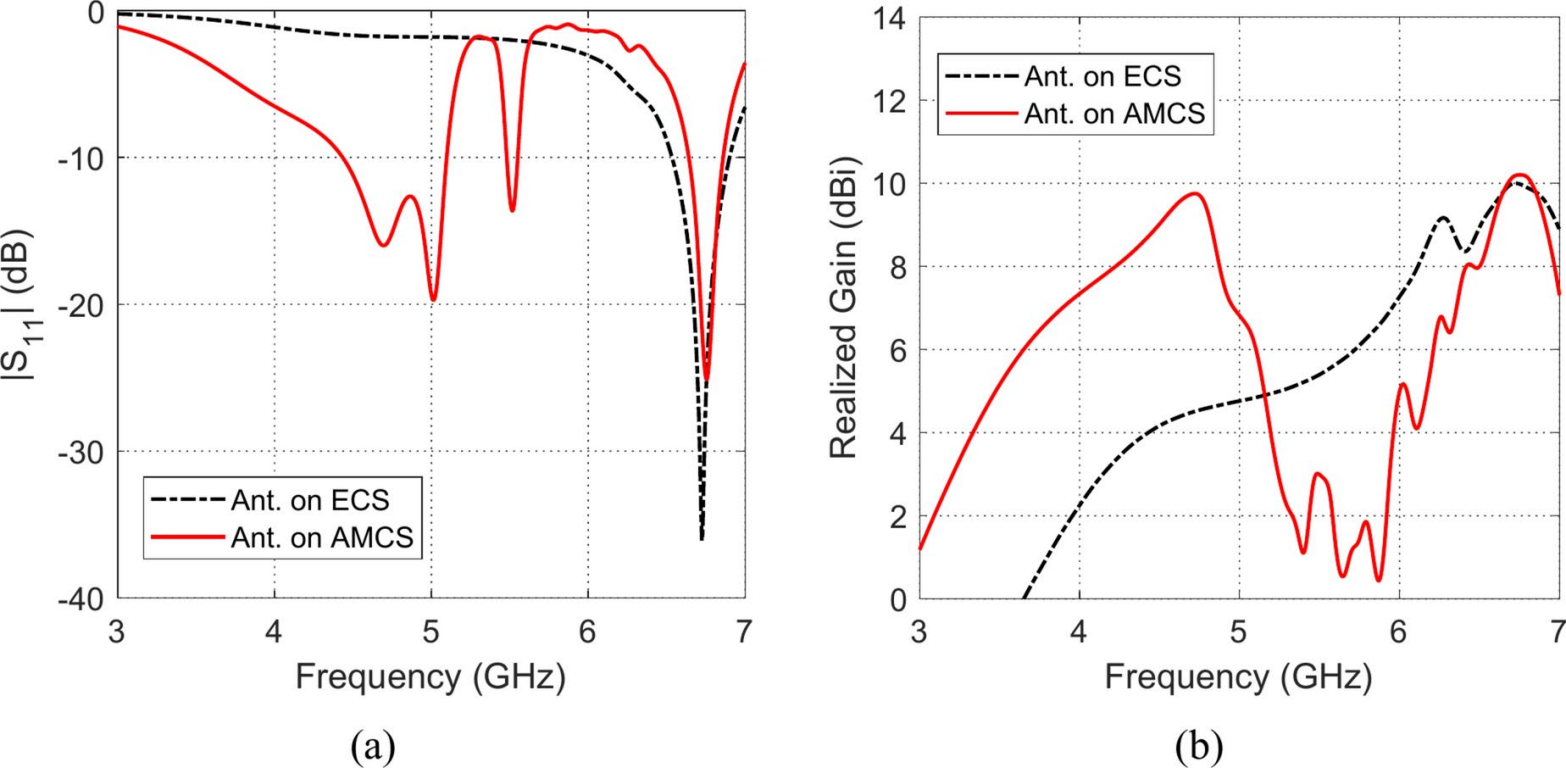
Frequency responses of (**a**) $\left|{S}_{11}\right|$ and (**b**) realized gain when the antenna is placed at height $D=1 \text{mm}$ over an ECS of dimensions $14\text{ cm}\times 14 \text{cm}$ and AMCS of $5\times 5$ cells.

**Fig. 28:**
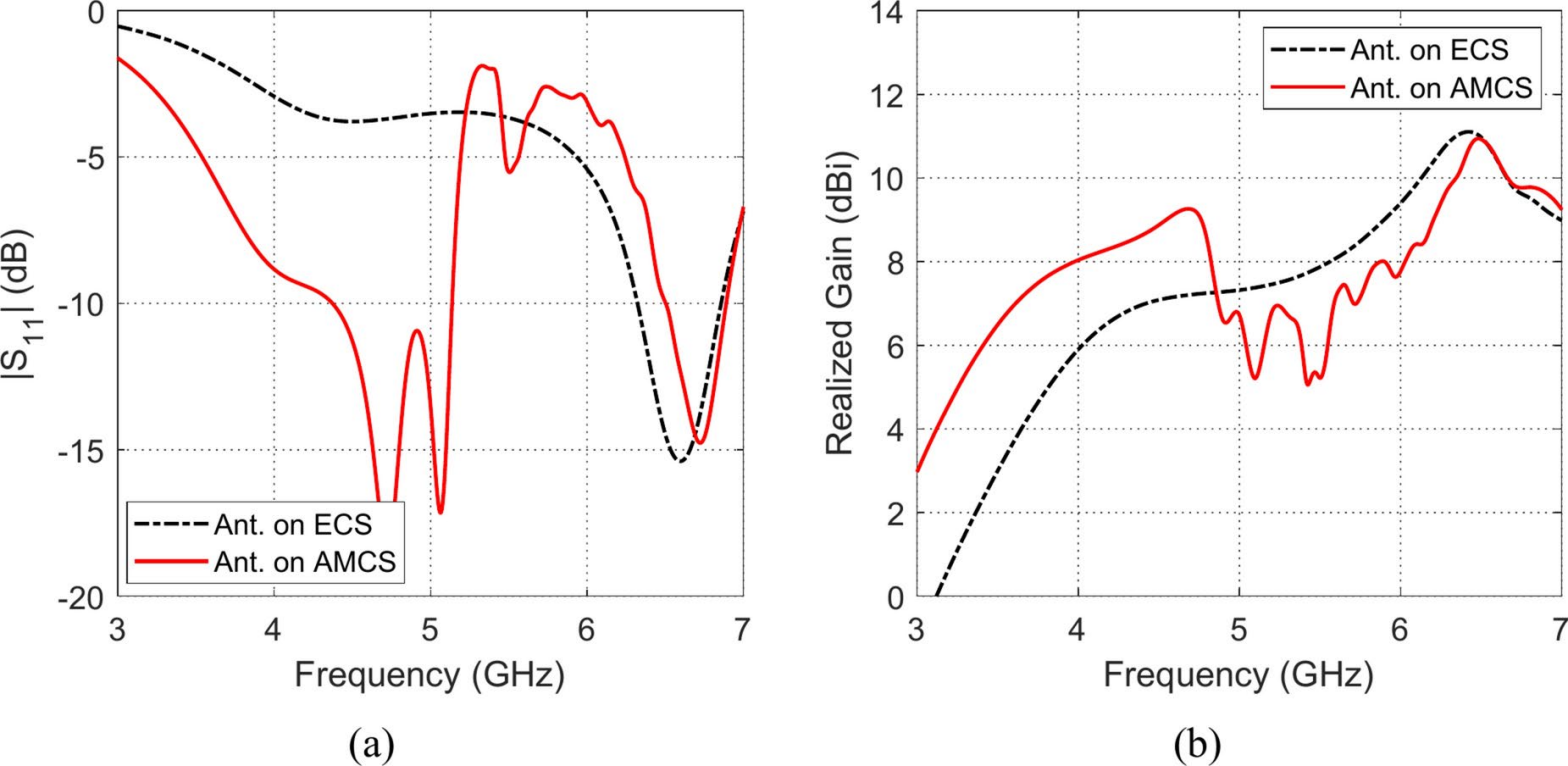
Frequency responses of (**a**) $\left|{S}_{11}\right|$, and (**b**) realized gain, when the antenna is placed at height $D=3 \text{mm}$ over an ECS of dimensions $14\text{ cm}\times 14 \text{cm}$ and AMCS of $5\times 5$ cells.

**Fig. 29:**
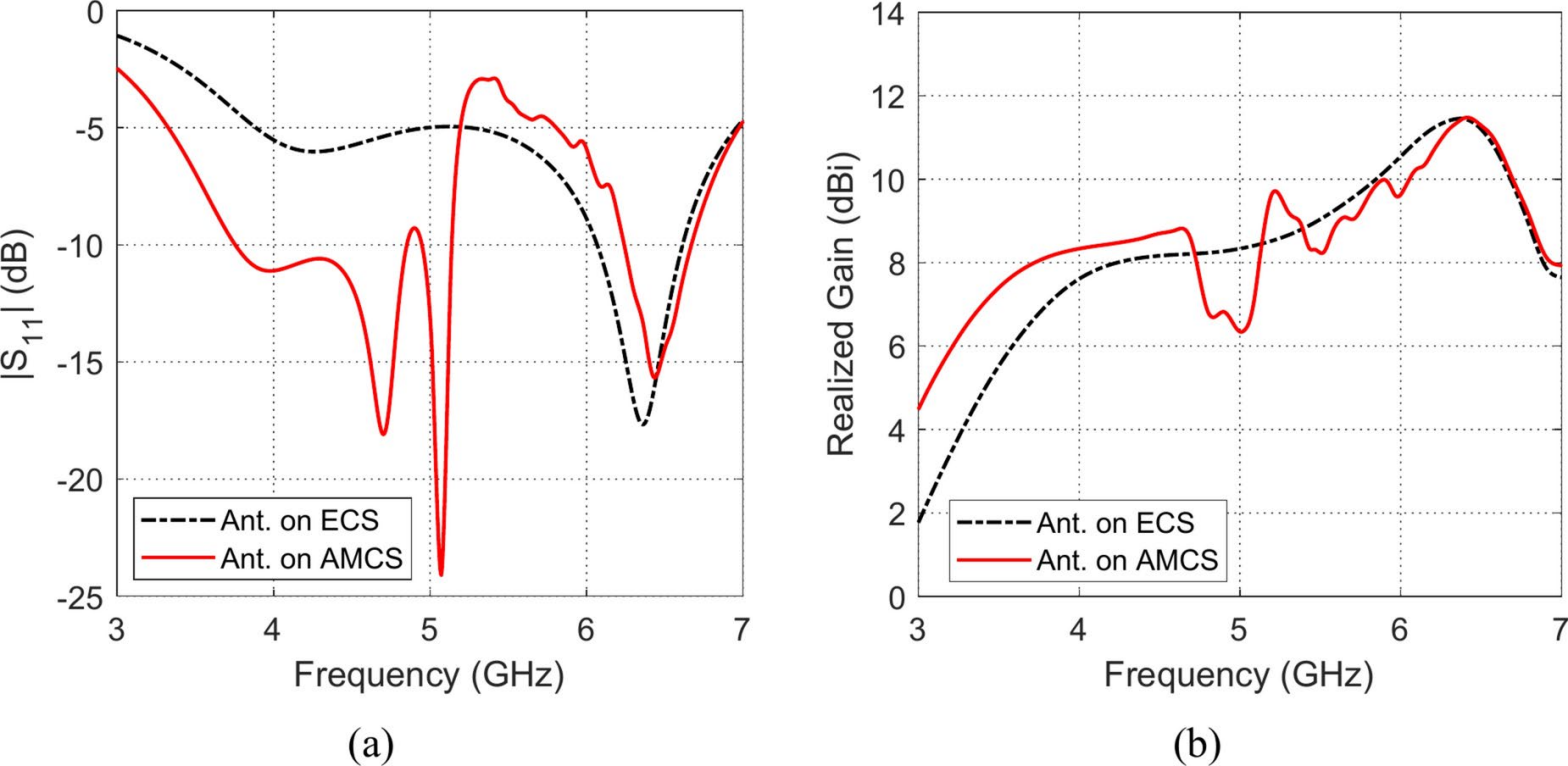
Frequency responses of (**a**) $\left|{S}_{11}\right|$, and (**b**) realized gain, when the antenna is placed at height $D=5 \text{mm}$ over an ECS of dimensions $14\text{ cm}\times 14 \text{cm}$ and AMCS of $5\times 5$ cells.

**Fig. 30:**
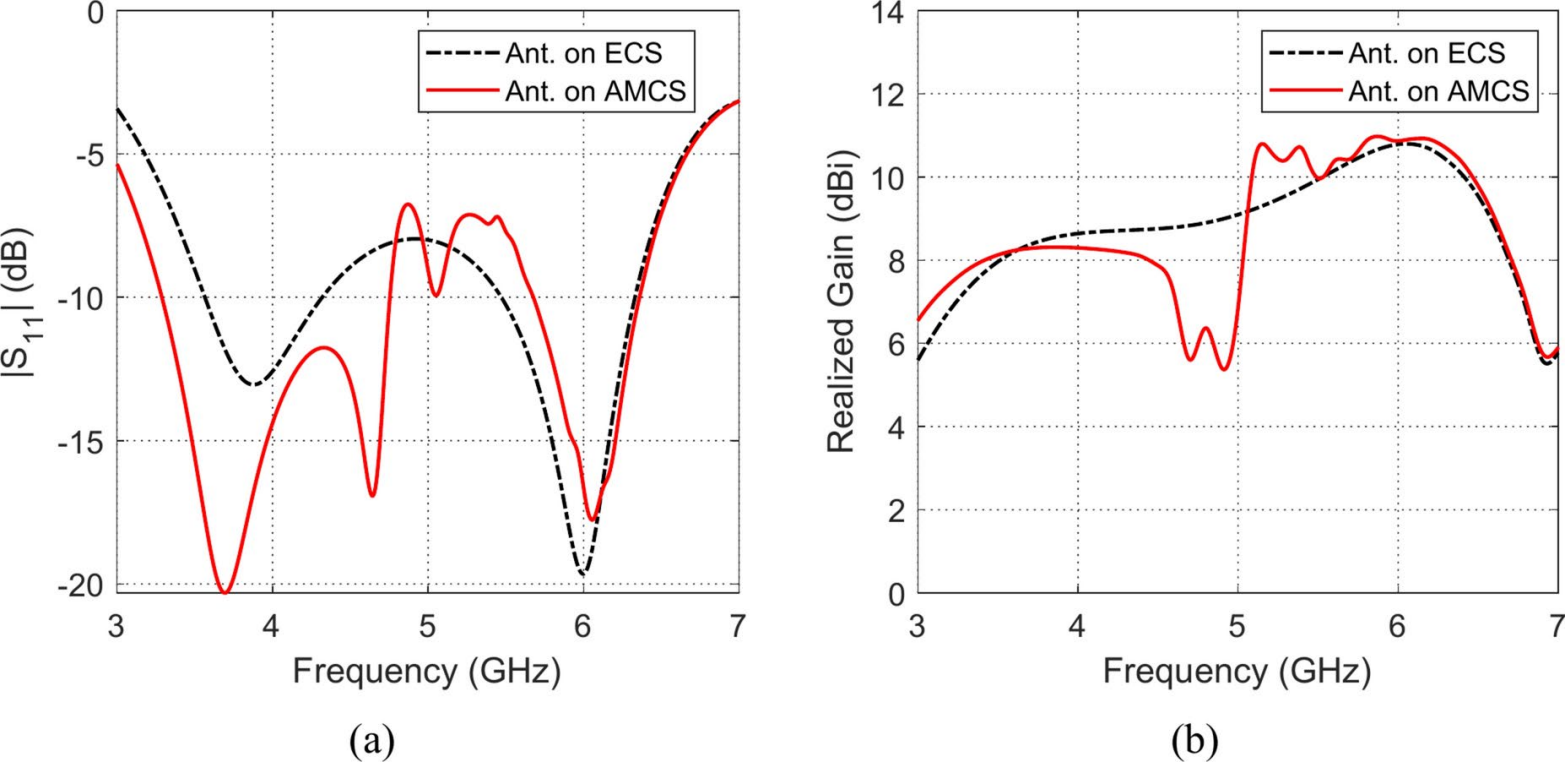
Frequency responses of (**a**) $\left|{S}_{11}\right|$, and (**b**) realized gain, when the antenna is placed at height $D=10 \text{mm}$ over an ECS of dimensions $14\text{ cm}\times 14 \text{cm}$ and AMCS of $5\times 5$ cells.

**Fig. 31:**
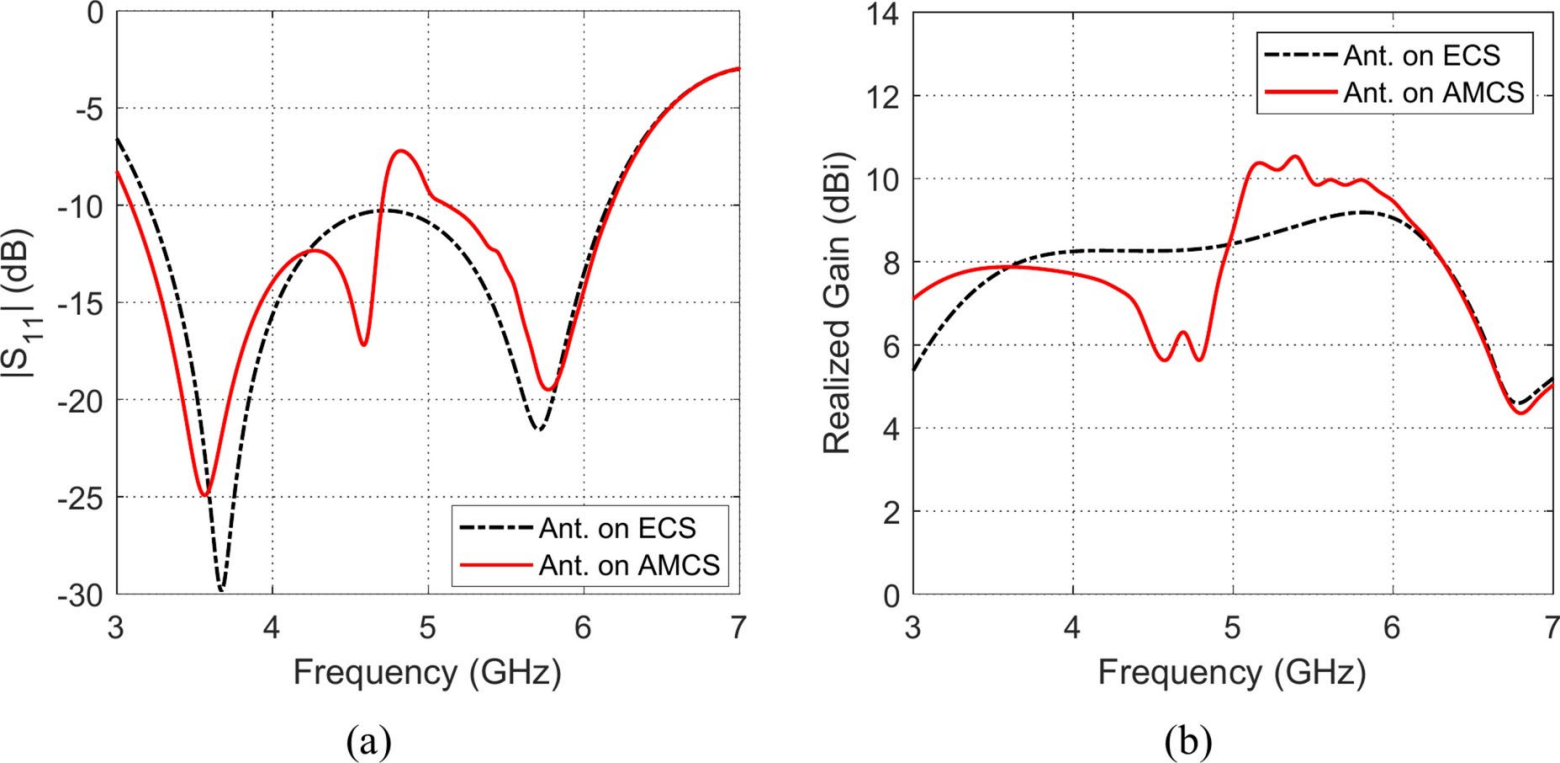
Frequency responses of (**a**) $\left|{S}_{11}\right|$, and (**b**) realized gain, when the antenna is placed at height $D=15 \text{mm}$ over an ECS of dimensions $14\text{ cm}\times 14 \text{cm}$ and AMCS of $5\times 5$ cells.

**Fig. 32:**
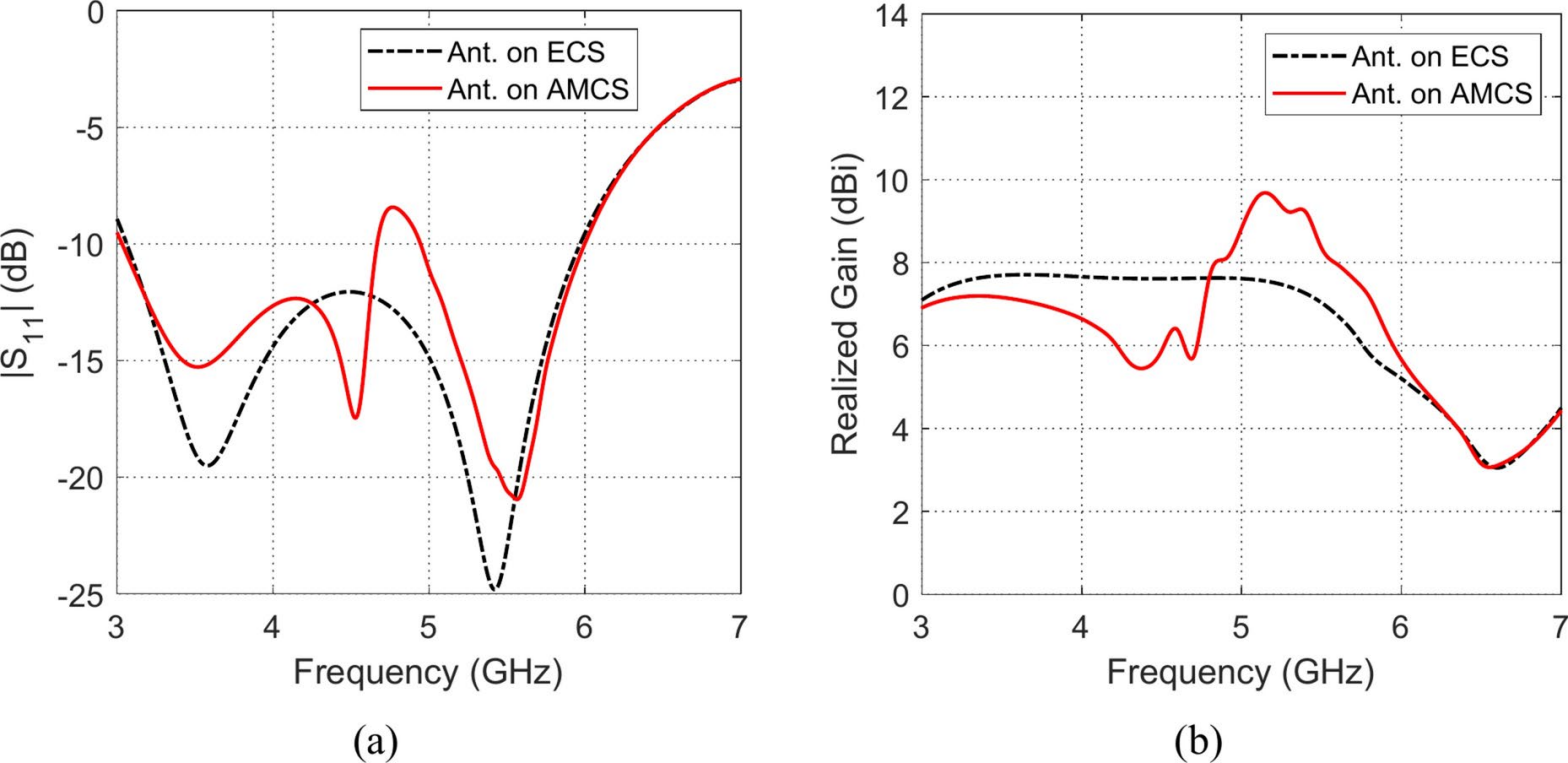
Frequency responses of (**a**) $\left|{S}_{11}\right|$, and (**b**) realized gain, when the antenna is placed at height $D=20 \text{mm}$ over an ECS of dimensions $14\text{ cm}\times 14 \text{cm}$ and AMCS of $5\times 5$ cells.

The effects of placing the omnidirectional planar octagon-shaped antenna at different heights over the AMCS (described in Sections “AMC reflector design” and “Performance of the monopole patch antenna when placed over AMCS”) and an ECS of the same dimensions on the impedance matching frequency band and the realized gain are depicted in Figs. 27, 28, 29, 30, 31, and 32. It is shown that for $D\le 10 \text{mm}$ (i.e. $D\le {\lambda }_{0}/4; {\lambda }_{0}$ is the resonant wavelength of the AMCS, which ids the wavelength at which the reflection from the AMCS has a phase of $0^\circ$), the placement of the ECS below the antenna produces has bad effect on the antenna impedance matching and results in a very narrow frequency band. However, for the $D\le 10 \text{mm}$, the placement of the AMCS results in multiple frequency bands of impedance matching. On the other hand, it is shown that, for $D>10 \text{mm}$ (i.e. $D>{\lambda }_{0}/4$), the placement of the ECS below the antenna in a wideband continuous frequency band of impedance matching. Thus, for $D>10 \text{mm}$, the ECS is superior to the AMCS regarding to the impedance matching bandwidth.

On the other hand, for $D\le 10 \text{mm}$, it is shown that the realized gain obtained at the lower frequencies when the AMCS is employed is much higher than that obtained when the ECS is employed. However, at the higher frequencies, it is shown that the realized gain produced by employing the ECS is higher than that produced by employing the AMCS. On the other side, for values of $D>10 \text{mm}$ (i.e. $D>{\lambda }_{0}/4$), the employment of the ECS gives a realized that is higher than that produced by the AMCS for the lower frequencies whereas the AMCS seems to be superior to the ECS at the higher frequencies at it gives higher values of the realized gain.

### Radiation and total efficiencies

**Fig. 33:**
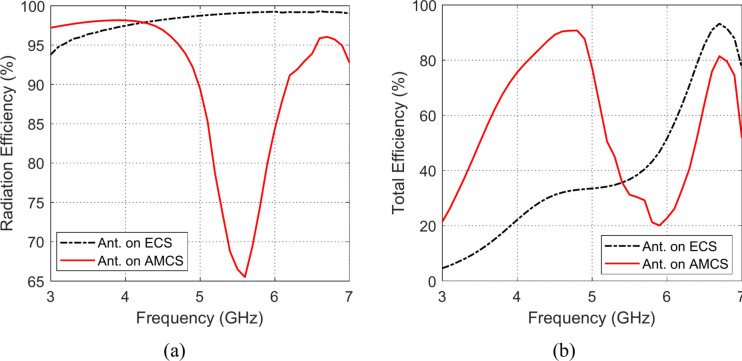
Variation of the (**a**) radiation efficiency and (**b**) total efficiency, with the frequency when the antenna is placed at height $D=1 \text{mm}$ over an ECS of diemnsions $14\text{ cm}\times 14 \text{cm}$ and AMCS of $5\times 5$ cells.

**Fig. 34:**
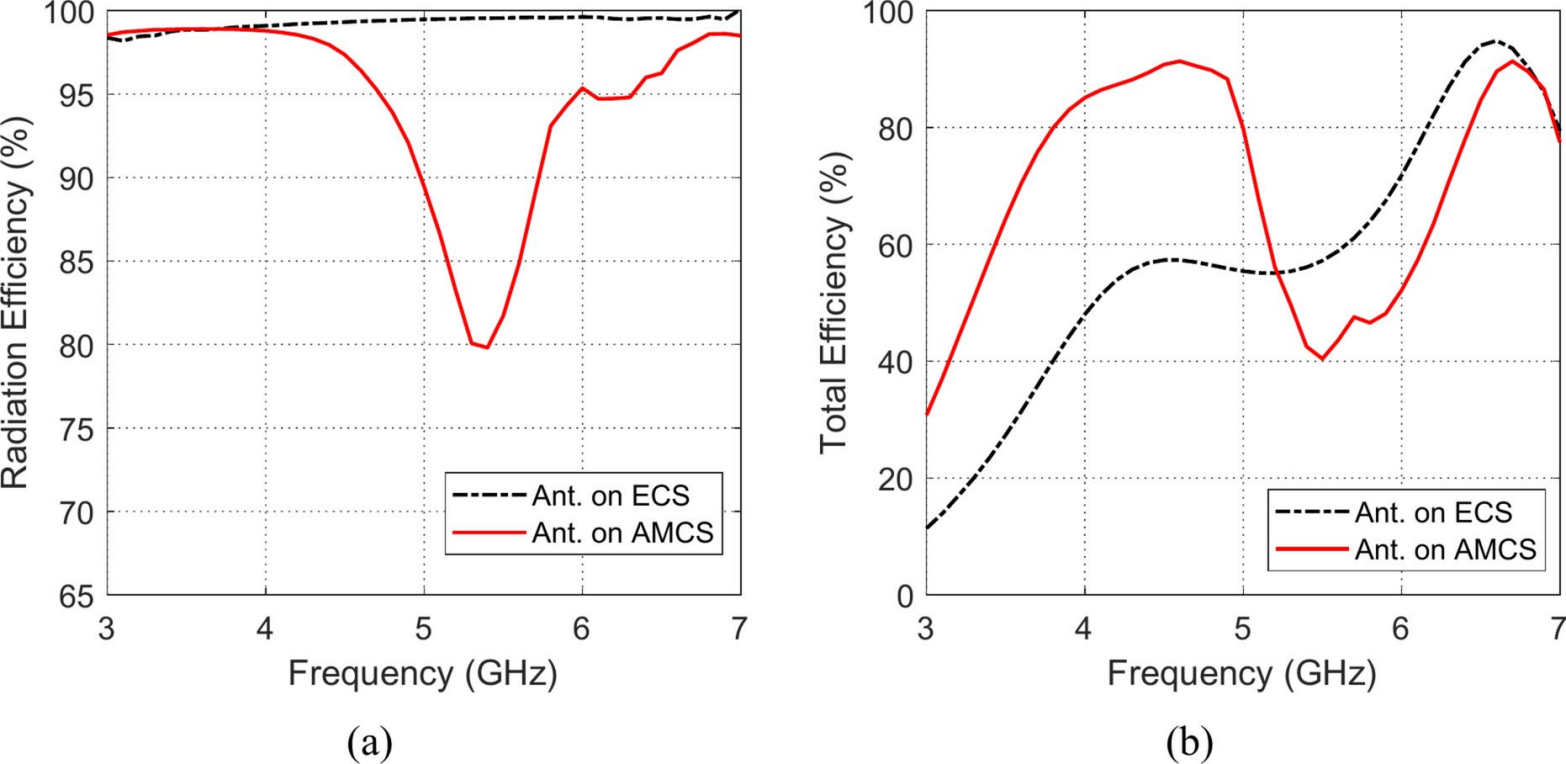
Variation of the (**a**) radiation efficiency and (**b**) total efficiency, with the frequency when the antenna is placed at height $D=3 \text{mm}$ over an ECS of diemnsions $14\text{ cm}\times 14 \text{cm}$ and AMCS of $5\times 5$ cells.

**Fig. 35:**
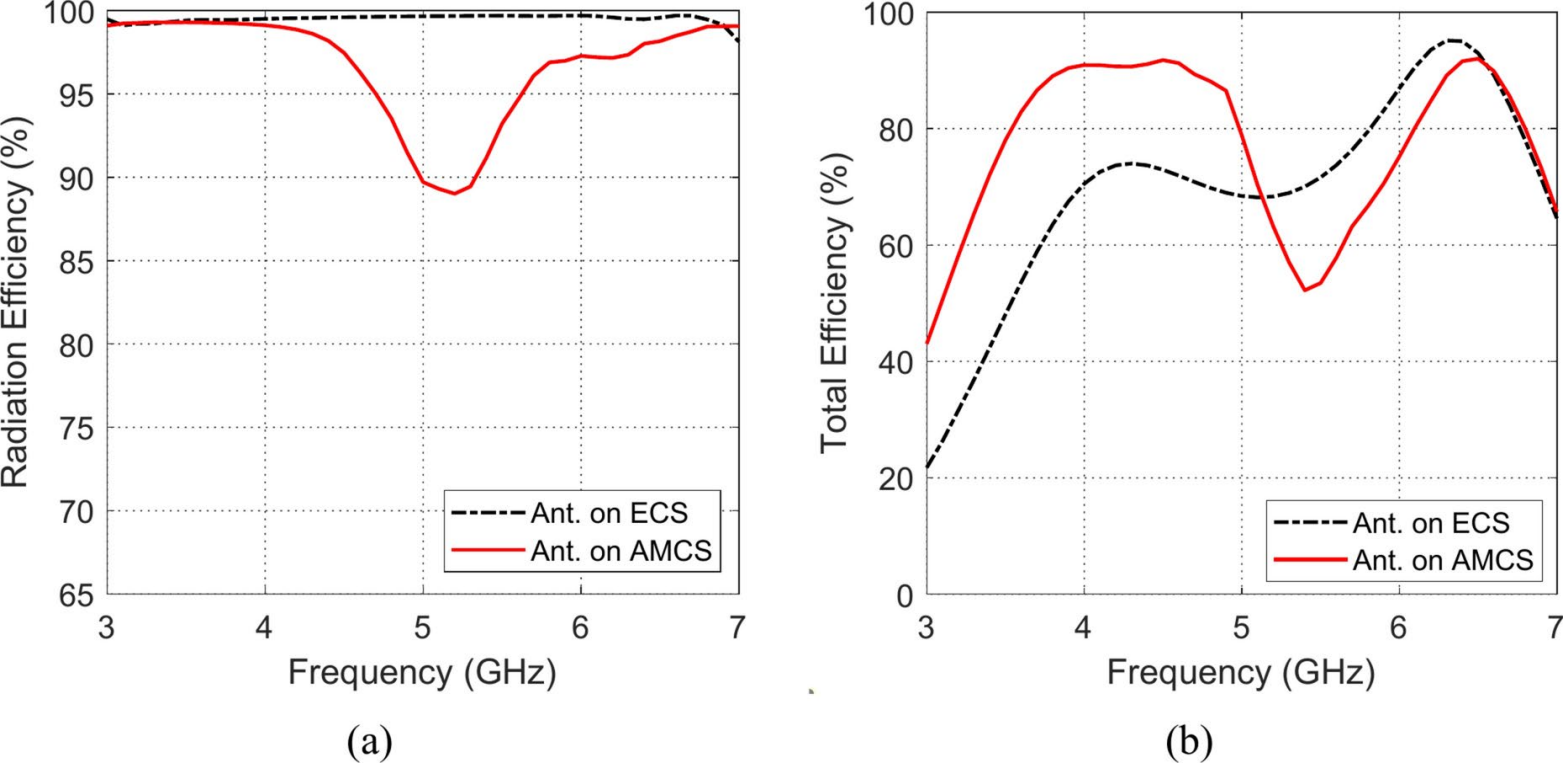
Variation of the (**a**) radiation efficiency and (**b**) total efficiency, with the frequency when the antenna is placed at height $D=5 \text{mm}$ over an ECS of diemnsions $14\text{ cm}\times 14 \text{cm}$ and AMCS of $5\times 5$ cells.

**Fig. 36:**
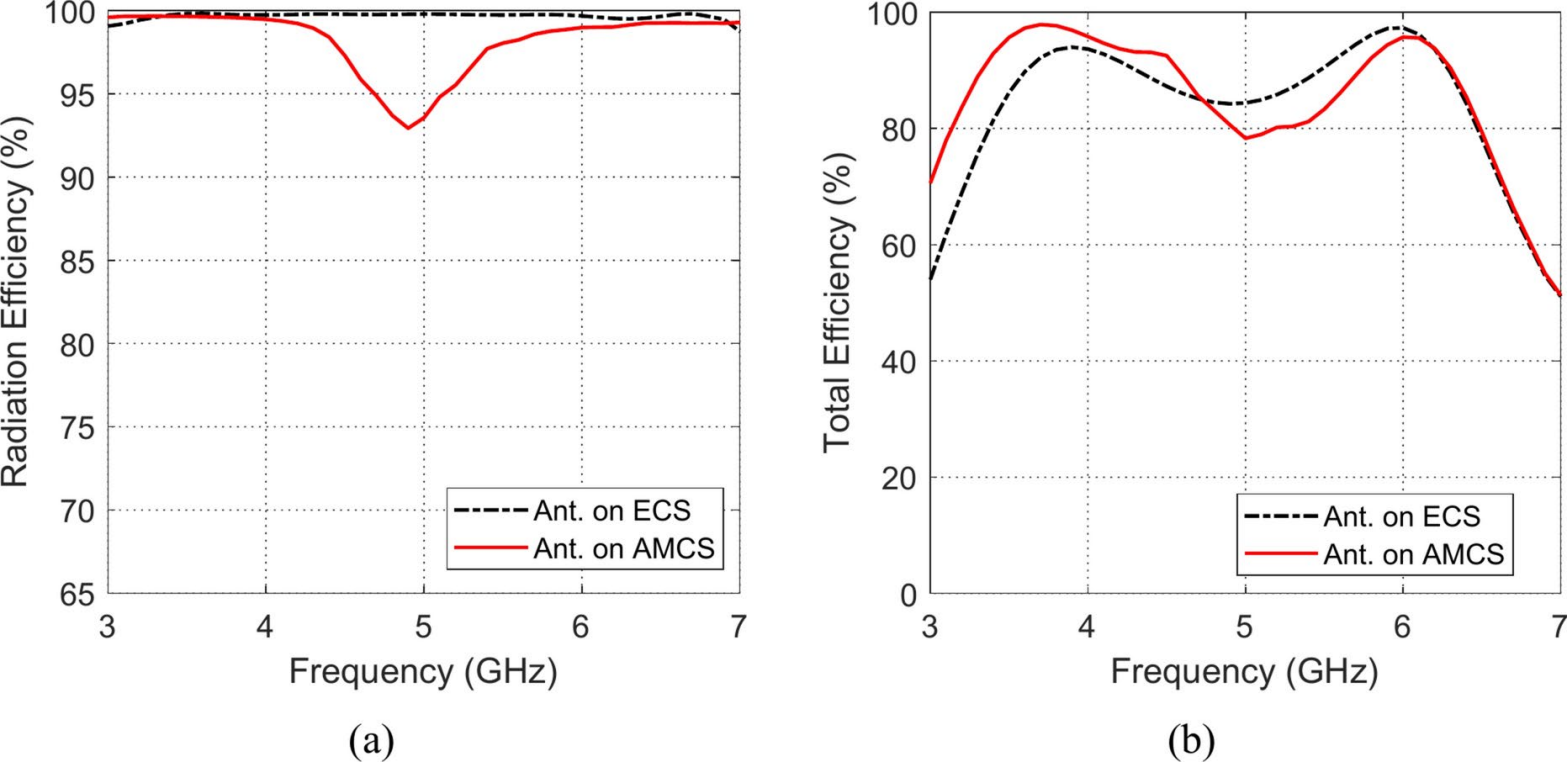
Variation of the (**a**) radiation efficiency and (**b**) total efficiency, with the frequency when the antenna is placed at height $D=10 \text{mm}$ over an ECS of diemnsions $14\text{ cm}\times 14 \text{cm}$ and AMCS of $5\times 5$ cells.

**Fig. 37:**
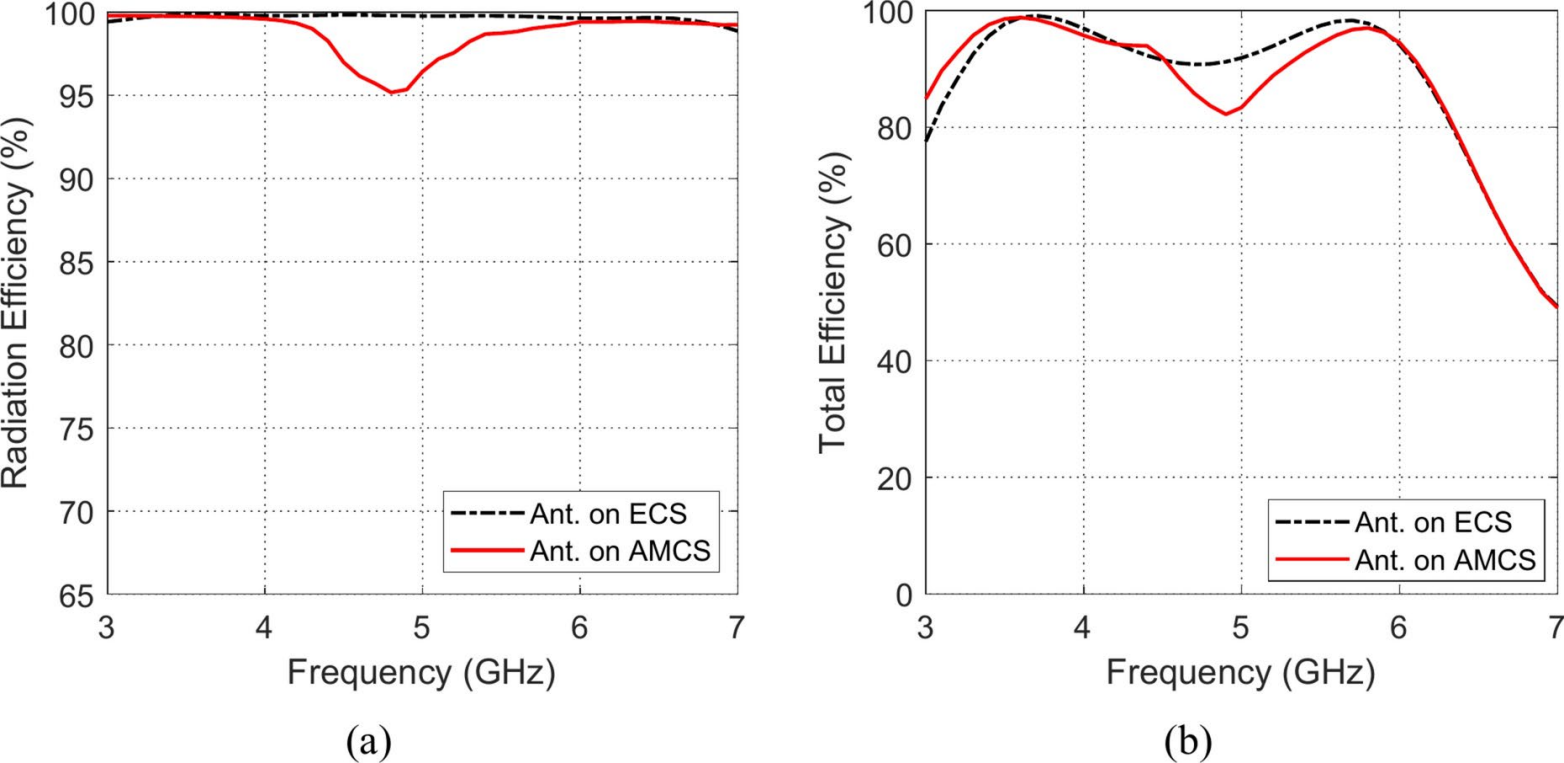
Variation of the (**a**) radiation efficiency and (**b**) total efficiency, with the frequency when the antenna is placed at height $D=15 \text{mm}$ over an ECS of diemnsions $14\text{ cm}\times 14 \text{cm}$ and AMCS of $5\times 5$ cells.

**Fig. 38:**
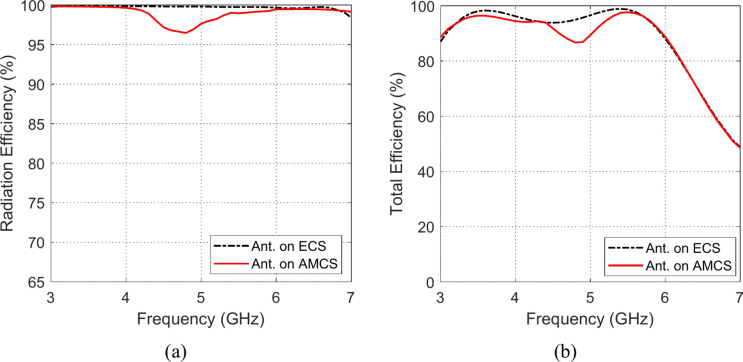
Variation of the (**a**) radiation efficiency and (**b**) total efficiency, with the frequency when the antenna is placed at height $D=20 \text{mm}$ over an ECS of diemnsions $14\text{ cm}\times 14 \text{cm}$ and AMCS of $5\times 5$ cells.

The frequency responses of the radiation and total efficiencies of the radiating structures that are composed of the planar monopole antenna when placed at different heights over the AMCS and the ECS reflectors are presented in the Figs. 33, 34, 35, 36,37, and 38. It is shown that the radiation efficiency achieved in the case of employing the ECS is always greater than that achieved by employing the AMCS. However, this result is attributed to the neglecting the return loss at the antenna feeding port in the calculation of the radiation efficiency. Therefore, the total efficiency can be used as better indication of the antenna efficiency. It is shown that for $D\le 10 \text{mm}$ (i.e. $D\le {\lambda }_{0}/4; {\lambda }_{0}$ is the resonant wavelength of the AMCS), the total efficiency obtained at the lower frequencies when the AMCS is employed is much higher than that obtained when the ECS is employed. However, at the higher frequencies, it is shown that the total efficiency produced by employing the ECS is higher than that produced by employing the AMCS for $D\le 10 \text{mm}$. On the other side, for values of $D>10 \text{mm}$ (i.e. $D>{\lambda }_{0}/4$), the employment of both surfaces gives total efficiency that is greater than 80%, where the ECS seems to be superior to the AMCS as its gives higher total efficiency especially near the middle of the frequency band.

### Radiation patterns

**Fig. 39:**
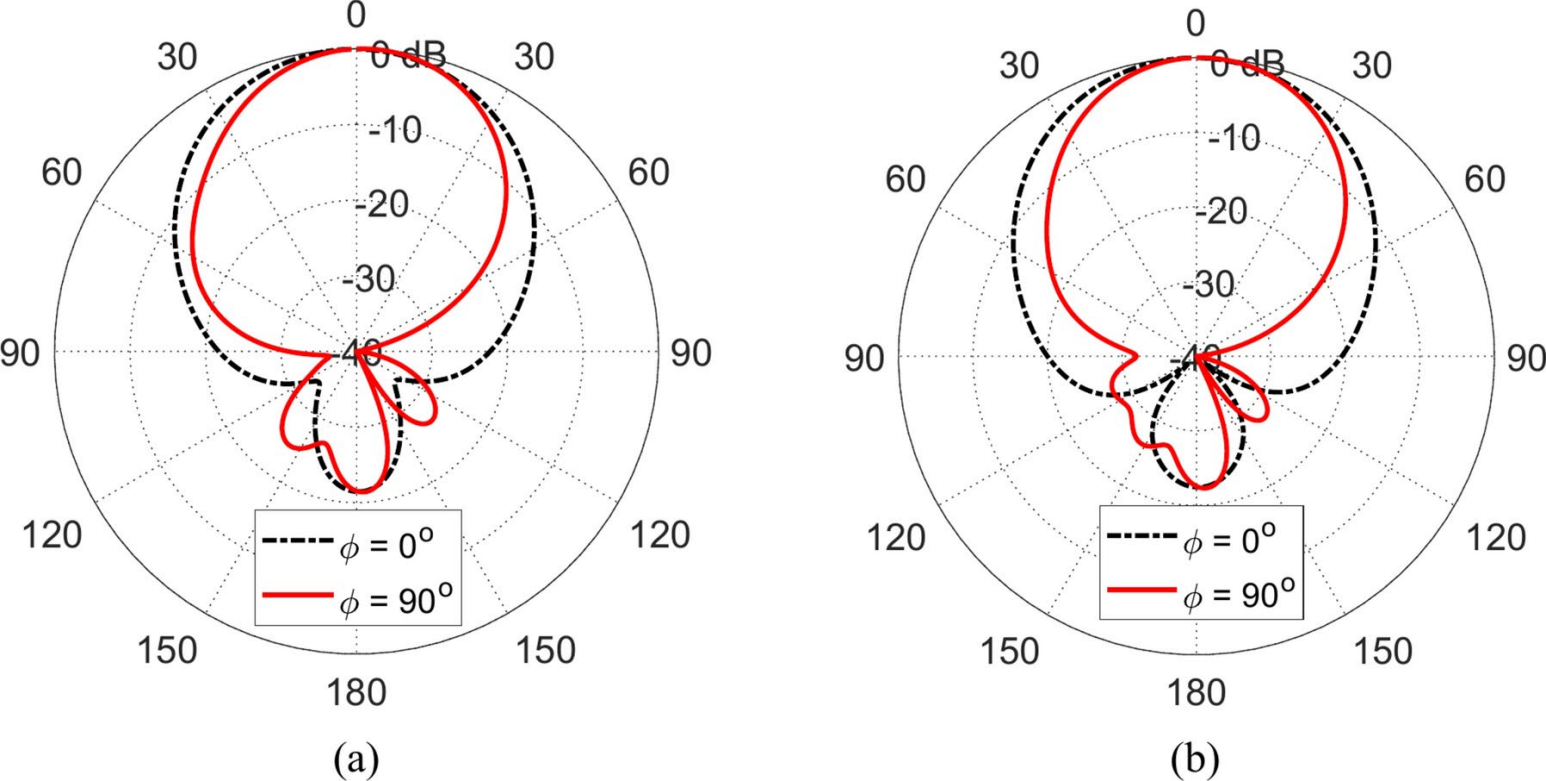
Radiation patterns obtained by simulation at $6.3\text{ GHz}$ when the proposed monopole patch antenna is placed at a height of $D=5\text{ mm}$ over (**a**) an AMCS of $5\times 5$ cells and dimensions $14 \text{cm}\times 14 \text{cm}$, and (**b**) a square ECS of dimensions $14 \text{cm}\times 14 \text{cm}$.

One of the interesting results is that when the planar octagon-shaped monopole antenna is placed at a height $D=5 \text{mm}$ above the proposed AMCS and an ECS of the same dimensions, the produced radiation patterns at $6.3\text{ GHz}$ are presented in Fig. 39a, b, respectively. The radiation patterns produced by employing the two surfaces seem to be similar. Moreover, employing any of the two surfaces as a reflector results in the same value of realized gain in the forward direction at $6.3 \text{GHz}$, which is $11.5 \text{GHz}$.

**Fig. 40:**
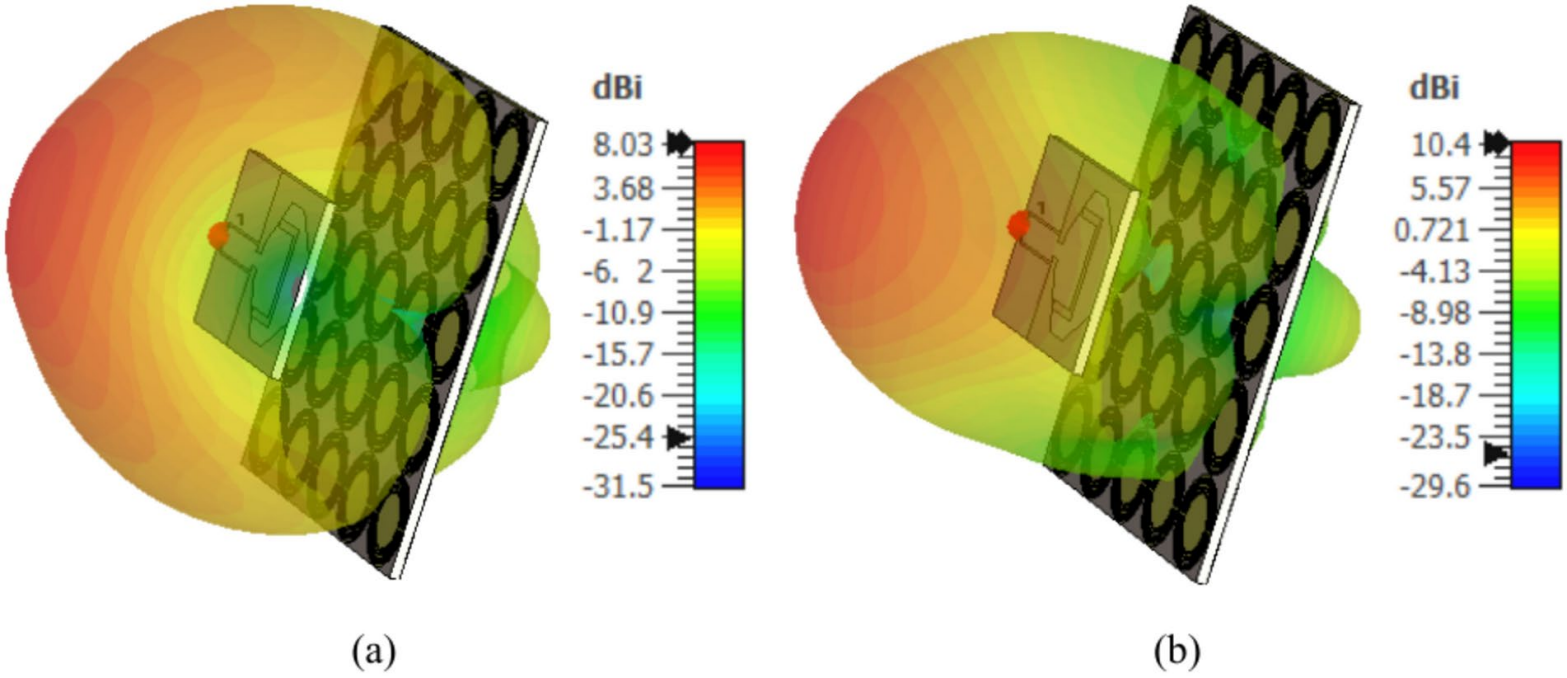
3D gain patterns produced by the monopole patch antenna when placed at a height $D=5\text{ mm}$ over an AMCS of $5\times 5$ cells and dimensions $14 \text{cm}\times 14 \text{cm}$ at (**a**) 4 GHz and (**b**) 6 GHz.

The 3D gain patterns produced by the monopole patch antenna when placed at a height $D=5\text{ mm}$ over an AMCS of $5\times 5$ cells and dimensions $14 \text{cm}\times 14 \text{cm}$ are presented in Fig. 40 at 4 GHz and 6 GHz. It is shown that, owing to the placement of the AMCS below the antenna. the radiation patterns are unidirectional with relatively high gain (8 dBi at 4 GHz and 10.4 dBi at 6 GHz.)

### Summarized comparison

A summary of the results of comparison between the antenna performance when the AMCS and the ECS are used for producing unidirectional radiation and enhancing the antenna gain are listed in Table 3. It is shown that, for all the values of $D$, the AMCS results in a multiband operation when used as a reflector whereas the ECS usually results in a single band operation. For values of $D\le 10 \text{mm}$ (i.e. $D\le {\lambda }_{0}/4; {\lambda }_{0}$ is the resonant wavelength of the AMCS), the AMCS is superior to the ECS as it results in wider frequency band. However, for values of $D>10 \text{mm}$ (i.e. $D>{\lambda }_{0}/4$), the ECS is superior to the AMCS as it results in wider frequency band and higher total efficiency.

**Table 3:** Comparison between the performance metrics when the AMCS and the ECS are placed at a distance $D=5 \text{mm}$ below the proposed antenna for producing unidirectional radiation.

Height (D) above Reflector	Antenna on ECS			Antenna on AMCS		
	Frequency band (GHz)	Realized gain (dBi)	Total efficiency (%)	Frequency band (GHz)	Realized gain (dBi)	Total efficiency (%)
$1\text{ m}$m	$6.5-6.9$	$10.1$	$93.3$	$4.5-5.1$	$9.5$	$90.8$
	$-$	$-$	$-$	$5.5-5.6$	$2.5$	$31.0$
	$-$	$-$	$-$	$6.6-6.9$	$10.2$	$80.0$
$3\text{ m}$m	$6.3-6.9$	$10.7$	$95$	$4.4-5.1$	$9.0$	$90.5$
	$-$	$-$	$-$	$6.5-6.9$	$10.0$	$91.3$
$5\text{ m}$m	$6.1-6.6$	$11.5$	$95$	$3.7-5.2$	$8.7$	$90.4$
	$-$	$-$	$-$	$6.2-6.7$	$11.5$	$91.0$
$10\text{ m}$m	$3.6-4.3$	$8.6$	$94$	$3.3-4.8$	$8.3$	$95.8$
	$5.5-6.3$	$10.9$	$97$	$5.7-6.4$	$10.9$	$95.7$
$15\text{ m}$m	$3.2-6.2$	$8.5$	$94$	$3.1-4.7$	$7.8$	$92.0$
	$-$	$-$	$-$	$5.1-6.2$	$10.0$	$93.4$
$20\text{ mm}$	$3.0-6.0$	$7.6$	$97$	$3.0-4.7$	$6.8$	$94.5$
	$-$	$-$	$-$	$5.0-6.0$	$8.6$	$95.7$

## Summary of the proposed antenna performance

The most important performance metrics of the proposed radiating structure including the dimensions, the AMCS bandwidth, and the maximum gain are compared to those achieved in other recently published related work. As shown in Table 4, the proposed work comes in the context of enhancing the performance of the wideband omnidirectional antennas by means of AMCS. It is shown from the comparison the proposed radiating structure has intermediate size among those listed in Table 4 with relatively wide frequency band and high gain when compared to most of the other published designs.

**Table 4:** Comparison with other published designs of wideband antennas based on wideband AMCS.

Work	Antenna dimensions ($\text{mm}\times \text{mm}\times \text{mm}$)	Overall dimension	With AMC bandwidth GHz (%)	Without/with AMC maximum gain (dBi)
^4^	$25\times 20\times 3.2$	$56\times 56\times 16.2$	$44.6\%$	3–7.91
^10^	$30\times 32\times 1.6$	$30\times 32\times 4.8$	6.5–8.3(24.32%)	4.25–6.25
^15^	$68\times 68\times 1.6$	$110\times 110\times 58$	4.5–6.5(24%)	4.5–$10$
^16^	$26\times 26\times 1.6$	$61\times 61\times 10$	3–11.05(118%)	7.8–9.6
^17^	$14.8\times 18\times 2.5$	$59\times 72\times 2.53$	60–63(4.8%)	12–15.6
Proposed	37 $\times 27$×1.57	$70 \times 70 \times 5.52$	5.27–9.61 GHz (58%)	3.5–$12.0$

## Conclusion

A wideband planar antenna and an AMCS have been proposed to produce unidirectional radiation with high gain over a wide frequency band. The proposed antenna is a planar octagon-shaped monopole patch with inverted U-slot and is fed through a coplanar waveguide (CPW). The AMCS is a metasurface constructed as periodic structure to produce reflection with 0° phase. This allows the antenna to be placed near the AMCS without destructive interference. Both the radiating patch and the feeding line are printed on a single-sided substrate of type Rogers RT5880 of dimensions $27 \text{mm}\times 37 \text{mm}$ and thickness $1.57\text{ mm}$. The patch geometry has been designed to maximize the radiation efficiency by cutting an inverted U-shaped slot with long base The proposed AMCS consists of $5\times 5$ cells and has dimensions $70\text{ mm }\times 70\text{ mm}$. The AMCS cells are printed on the top face of a substrate of type Rogers’ RO4003C of thickness $1.52\text{ mm}$. Both the monopole patch antenna and the AMCS are fabricated for experimental evaluation of the performance of the radiating structure. It is shown by simulation and measurement that the combined structure of the antenna based on the AMCS produces realized gain of $11.5\text{ dBi}$ and total efficiency of greater than $80\%$ over the operational frequency band ($3.5-6.5\text{GHz}$).

## Declarations

### Data availability

The datasets used and/or analyzed during the current study available from the corresponding author on reasonable request.
